# Innate and adaptive nasal mucosal immune responses following experimental human pneumococcal colonization

**DOI:** 10.1172/JCI128865

**Published:** 2019-09-16

**Authors:** Simon P. Jochems, Karin de Ruiter, Carla Solórzano, Astrid Voskamp, Elena Mitsi, Elissavet Nikolaou, Beatriz F. Carniel, Sherin Pojar, Esther L. German, Jesús Reiné, Alessandra Soares-Schanoski, Helen Hill, Rachel Robinson, Angela D. Hyder-Wright, Caroline M. Weight, Pascal F. Durrenberger, Robert S. Heyderman, Stephen B. Gordon, Hermelijn H. Smits, Britta C. Urban, Jamie Rylance, Andrea M. Collins, Mark D. Wilkie, Lepa Lazarova, Samuel C. Leong, Maria Yazdanbakhsh, Daniela M. Ferreira

**Affiliations:** 1 Department of Clinical Sciences, Liverpool School of Tropical Medicine, Liverpool, United Kingdom.; 2 Department of Parasitology, Leiden University Medical Center, Leiden, Netherlands.; 3 Bacteriology Laboratory, Butantan Institute, Sao Paulo, Brazil.; 4 Royal Liverpool and Broadgreen University Hospital, Liverpool, United Kingdom.; 5 Division of Infection and Immunity and; 6 Centre for Inflammation and Tissue Repair, University College London, London, United Kingdom.; 7 Malawi-Liverpool-Wellcome Trust Clinical Research Programme, Blantyre, Malawi.; 8 Department of Parasitology, Liverpool School of Tropical Medicine, Liverpool, United Kingdom.; 9 Aintree University Hospital NHS Foundation Trust, Liverpool, United Kingdom.; 10 Department of Otorhinolaryngology – Head and Neck Surgery, Aintree University Hospital NHS Foundation Trust, Liverpool, United Kingdom.

**Keywords:** Immunology, Infectious disease, B cells, Bacterial infections

## Abstract

*Streptococcus pneumoniae* (Spn) is a common cause of respiratory infection, but also frequently colonizes the nasopharynx in the absence of disease. We used mass cytometry to study immune cells from nasal biopsy samples collected following experimental human pneumococcal challenge in order to identify immunological mechanisms of control of Spn colonization. Using 37 markers, we characterized 293 nasal immune cell clusters, of which 7 were associated with Spn colonization. B cell and CD161^+^CD8^+^ T cell clusters were significantly lower in colonized than in noncolonized subjects. By following a second cohort before and after pneumococcal challenge we observed that B cells were depleted from the nasal mucosa upon Spn colonization. This associated with an expansion of Spn polysaccharide–specific and total plasmablasts in blood. Moreover, increased responses of blood mucosa-associated invariant T (MAIT) cells against in vitro stimulation with pneumococcus prior to challenge associated with protection against establishment of Spn colonization and with increased mucosal MAIT cell populations. These results implicate MAIT cells in the protection against pneumococcal colonization and demonstrate that colonization affects mucosal and circulating B cell populations.

## Graphical abstract

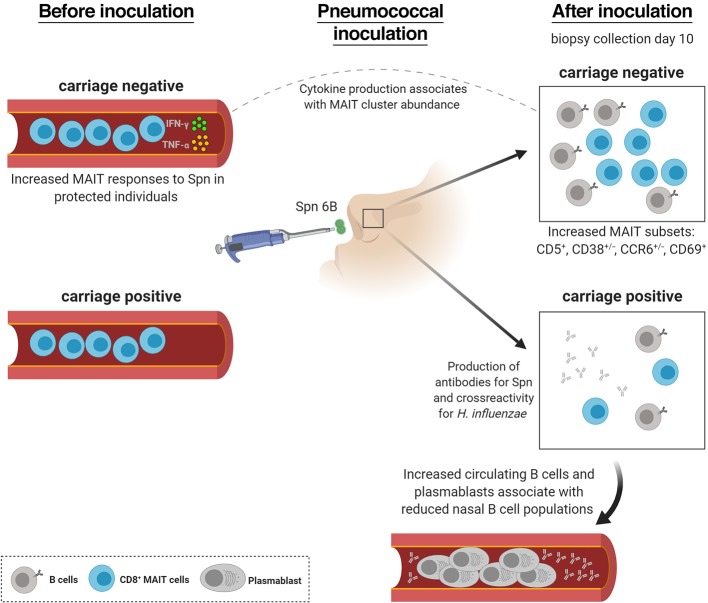

## Introduction

*Streptococcus pneumoniae* (Spn) is a major cause of morbidity and mortality worldwide (1, 2). It is the most common bacterial cause of otitis media, pneumonia, and meningitis in children (1). Risk factors for pneumococcal disease include very young or advanced age, coinfection with influenza, HIV infection, chronic lung disease, asplenia, and smoking (3).

However, nasopharyngeal colonization, or carriage, of Spn in the absence of disease is common, with approximately 50% of infants and 10% of adults colonized at any time (4). Carriage is an immunizing event in both children and adults but is also important as a prerequisite of disease and as the source of transmission (5–8). Successful colonization by Spn depends on many factors including bacterial factors, niche competition with other microbes, evasion of mucociliary clearance, and host nutrient availability as well as immunological control of Spn (9). Epidemiological and modeling data have demonstrated that the immunizing effect of carriage is likely mediated by a combination of serotype-dependent and serotype-independent mechanisms (10–12).

The introduction of pneumococcal conjugate vaccines (PCVs) has led to significant reductions in carriage prevalence of covered serotypes, leading to herd protection and a decrease in pneumococcal disease in unvaccinated adults in addition to conferring direct protection (13). However, only 13 of approximately 100 Spn serotypes are currently covered by PCVs and the elucidation of immune mechanisms that associate with the control of Spn carriage remains an area of active investigation (14).

Mouse models have suggested that Th17-mediated recruitment of neutrophils and monocytes to the nasopharynx is the mechanism of control and clearance of Spn carriage (15–17). In contrast, depletion of B cells or CD8^+^ T cells did not impair the clearance of Spn in murine models (18, 19). Amplification of monocyte recruitment in an auto-feedback loop via CCL2 was found to be important for clearance, further supporting the role for these cells in control of carriage (20). Innate factors have also been implicated in murine models as disruption of interferon α (IFN-α) or IL-1 signaling is associated with increased colonization (21, 22). Recently, we demonstrated using an experimental human pneumococcal challenge (EHPC) model that carriage leads to degranulation of nasal-resident neutrophils and recruitment of monocytes to the nasal mucosal surface (23). These responses were impaired by coinfection with live attenuated influenza virus, which associated with increased carriage density (24). Protection against experimental carriage acquisition in an unvaccinated setting is further associated with the levels of circulating memory B cells, but not levels of IgG, directed against the Spn polysaccharide capsule (25). Following PCV, very high levels of IgG associate with protection against experimental carriage acquisition, likely by mediating Spn agglutination followed by mucociliary clearance (26, 27). However, the relative role of these and other adaptive and innate immune cell subsets in controlling Spn at the human nasal mucosa remains largely unknown (28). The relatively small number of cells that can be collected from the nasal mucosa using minimally invasive nasal curettage has limited the capacity to analyze the role of cellular subsets in controlling Spn carriage at the human nasal mucosa (29).

Here, we collected nasal biopsies under local anaesthesia following experimental human pneumococcal challenge. This allowed for a comprehensive analysis of mucosal immunity during Spn carriage, as these samples yield substantially more cells than minimally invasive curettes. Nasal mucosal samples were analyzed using mass cytometry (CyTOF), a technique in which antibodies are labeled with rare-earth metals and that enabled the investigation of 37 protein markers simultaneously on a single-cell level (30). This method is ideally suited to investigate the relatively understudied mucosal immune populations, as the large number of markers allow the identification of previously unknown cell subsets and markers. Indeed, CyTOF has recently provided new insights into alveolar macrophage subpopulations in the lung and innate lymphoid cell differentiation pathways in the gut (31, 32). By combining nasal biopsies and CyTOF, we were thus able to study in depth the immunological role of innate and adaptive cell subsets at the human nasal mucosa and their role during pneumococcal colonization.

## Results

### Characterization of nasal immune populations.

**Figure 1:**
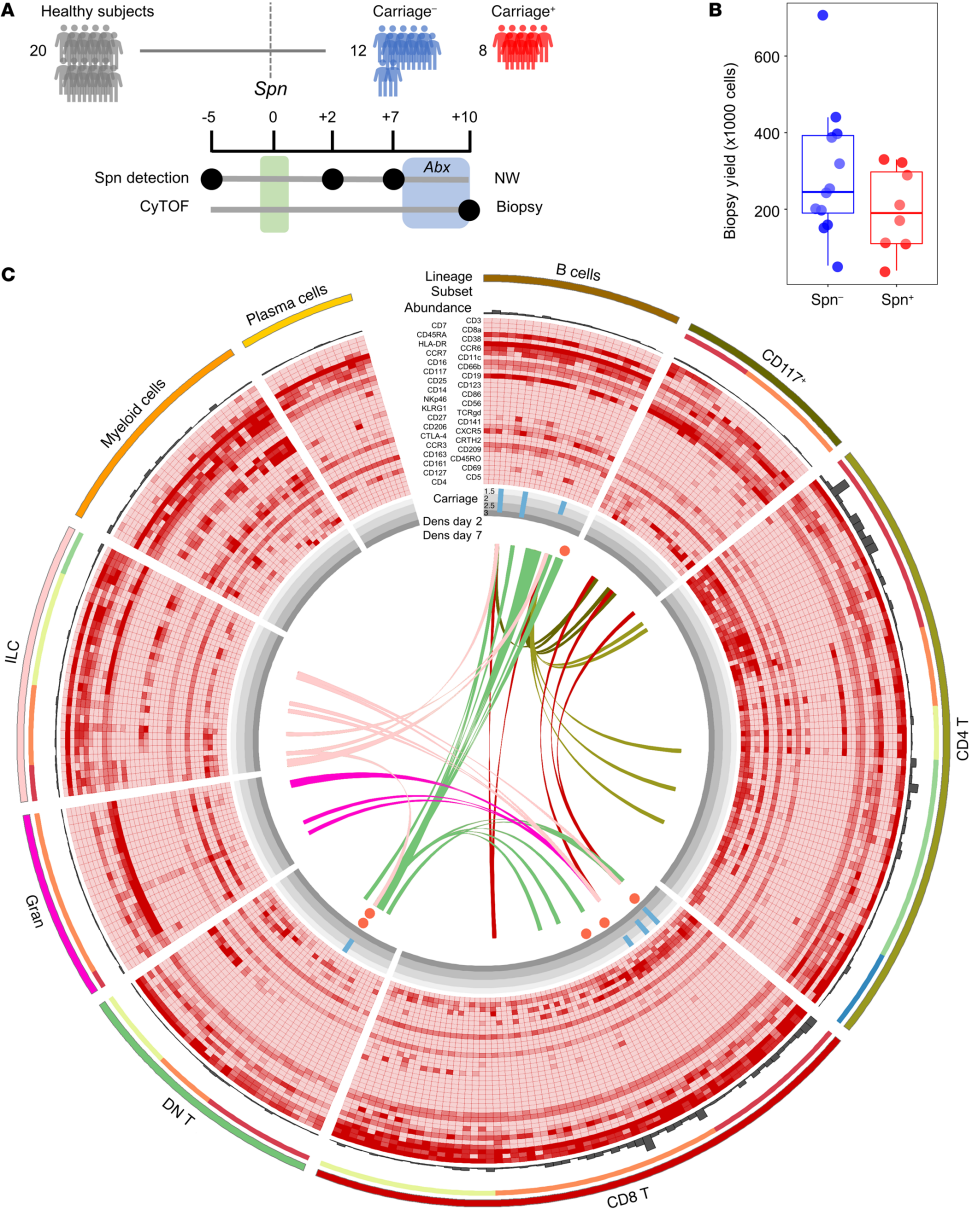
Mass cytometry from nasal biopsies following experimental human pneumococcal challenge. (**A**) Study design showing pneumococcal inoculation (green bar) and sample collection. Subjects who acquired pneumococcus following challenge are depicted in red (*n* = 8), while those protected are depicted in blue (*n* = 12). Antibiotics (Abx) were administered in the 3 days leading up to biopsy collection (blue area). NW, nasal wash. (**B**) Viable cell yield following enzymatic biopsy digestion for the 20 biopsies collected for CyTOF. Individual samples and box plots, depicting median and interquartile ranges, with whiskers extending to 1.5× interquartile range or maximum value, are shown. (**C**) Circle diagram showing all 293 defined clusters within 9 lineages and 22 subpopulations. From outside in: Number of cells in each cluster is depicted by gray bars. Relative expression for 36 markers is shown with red depicting higher expression (CD45 and Epcam are not depicted). Association with carriage state is shown, where blue bars depict the fold-change of the median of normalized abundance in carriage^–^ subjects over carriage^+^ subjects (Mann-Whitney test, *P* < 0.05 shown only). Significant correlations between Spn density on either day 2 or day 7 with normalized abundance are depicted by circles, with red indicating a negative association. Spearman’s correlation analyses were performed with all subjects, where carriage^–^ subjects were included with a density of 0. Ribbons connect highly correlated (*r* > 0.70) clusters that were associated with Spn carriage status not belonging to the same lineage, with color indicating the lineage of origin. DN T, double-negative T cells; Gran, granulocytes; ILC, innate lymphoid cells.

**Figure 2:**
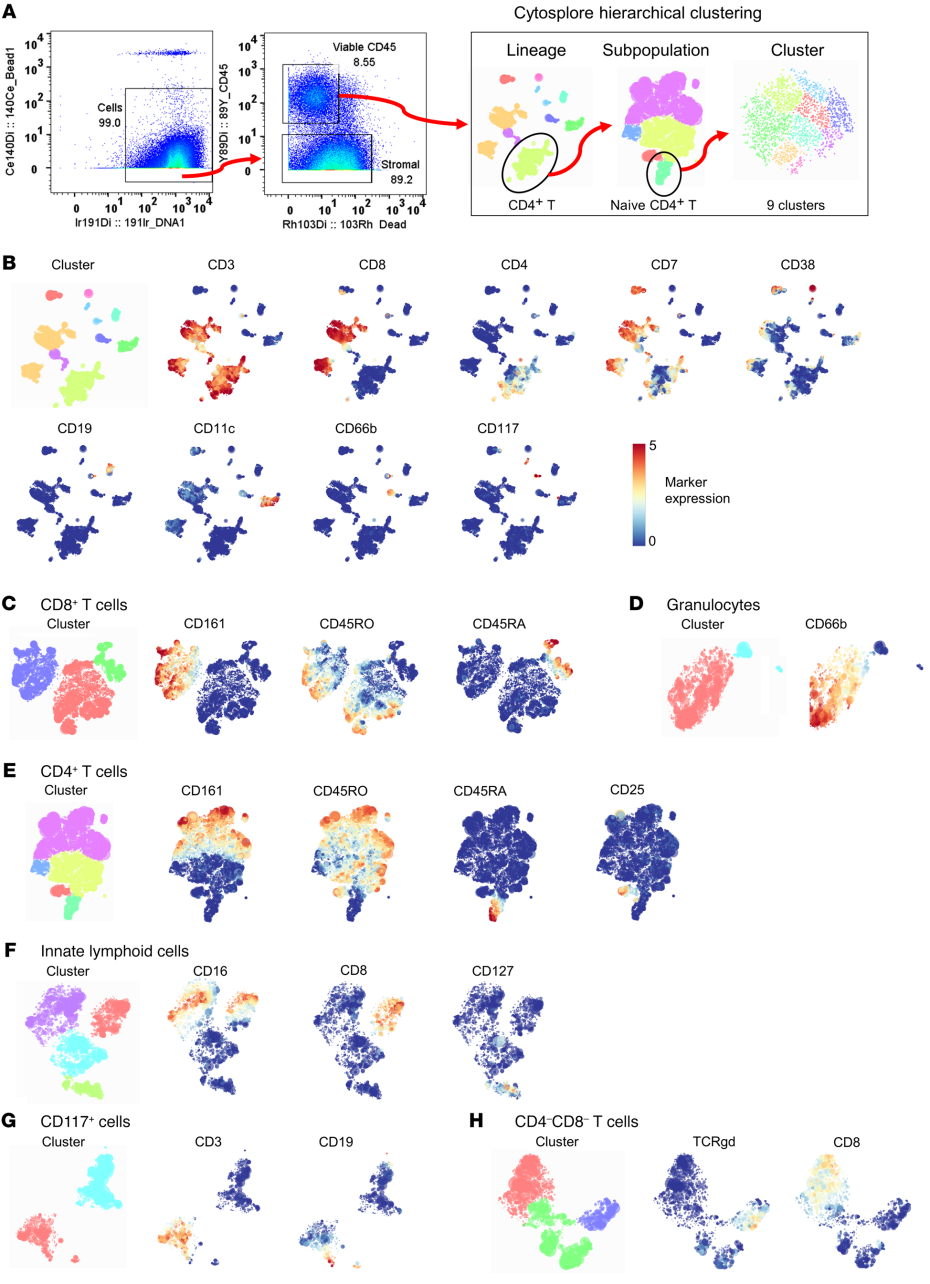
CyTOF analysis strategy. (**A**) CyTOF data files were pregated using FlowJo to identify cells (DNA^+^Bead^–^), followed by selecting viable immune cells (CD45^+^Dead^–^). These cells were exported and loaded in Cytosplore for hierarchical stochastic neighbor embedding (h-SNE), in which lineages, subpopulations, and clusters were sequentially identified in 3 steps. Gating for naive CD4^+^ T cells is shown as an example. (**B**) Cells were clustered using all 38 markers minus the epithelial marker Epcam and lineages were then defined based on the expression of 9 markers. Clustered lineages and expression of included markers are shown. Subpopulations for (**C**) CD8^+^ T cells, (**D**) granulocytes, (**E**) CD4^+^ T cells, (**F**) innate lymphoid cells, (**G**) CD117^+^ cells, and (**H**) double-negative T cells were defined based on the expression of the depicted markers. B cells, plasma cells, and myeloid cells were not further divided into subpopulations due to lack of clear clustering by relevant markers. Cell subpopulations were then further divided into clusters using all 38 markers minus the epithelial marker Epcam.

Twenty healthy subjects negative for natural pneumococcal carriage at baseline screening were challenged intranasally with type 6B Spn (Figure 1A and Table 1). Carriage state was assessed at days 2 and 7 after challenge and a nasal biopsy was collected at 10 days after challenge (Supplemental Video 1; supplemental material available online with this article; https://doi.org/10.1172/JCI128865DS1), the time point at which Spn starts to be cleared from the nose (33, 34). Eight subjects became colonized with Spn (carriage^+^), while 12 subjects remained carriage^–^ (Figure 1A). Biopsies yielded a median of 2.3 × 10^5^ cells (IQR: 1.6 × 10^5^ to 3.2 × 10^5^) per subject, approximately 90% of which were stromal cells, which were stained with a panel of 38 antibodies and analyzed by CyTOF (Figure 1B and Supplemental Table 1). Viable immune cells were manually gated from all acquired events and subsequently clustered by hierarchical stochastic neighbor embedding (h-SNE) using Cytosplore software (Figure 1C and Figure 2) (35–37). h-SNE is a recently developed method in which t-distributed stochastic neighbor embedding (t-SNE) is performed sequentially to first cluster global cell populations, each of which is then in turn clustered into subpopulations.

Based on the expression of 37 markers, a total of 199,426 immune cells from all subjects were divided into 9 lineages (CD8^+^ T cells, CD4^+^ T cells, myeloid cells, innate lymphoid cells, B cells, double-negative T cells, granulocytes, CD117^+^ cells, and plasma cells, in order of decreasing abundance). These cell lineages were further divided into 22 subpopulations and 293 clusters (Figure 1C and Table 2). Cell numbers were normalized to the number of stromal cells for each subject to correct for varying biopsy yields. Normalized abundances were then compared between carriage^–^ and carriage^+^ subjects for each of the lineages, subpopulations, and clusters. There were no significant differences in frequencies between total lineages or subpopulations between carriage^–^ and carriage^+^ subjects. However, at a finer level 7 clusters were significantly higher in carriage^–^ than in carriage^+^ subjects (Figure 1C, blue bars). Of note, 3 B cell clusters were higher in carriage^–^ subjects (Figure 1C). Moreover, 3 CD8^+^ T cell clusters, all expressing CD161, and 1 CD8^dim^ T cell cluster were higher in carriage^–^ subjects than in carriage^+^ subjects (Figure 1C). The 7 significant clusters strongly correlated (*r* > 0.70) with 88 clusters in other lineages/subpopulations, 68 of which were in B or T cell lineages, highlighting an interconnectivity between B and T cell subpopulations in the human nasopharynx (Figure 1C). We also investigated whether Spn load was associated with cluster abundance to determine the effect of cell clusters on the control of bacterial load. At day 2, four clusters were significantly negatively correlated with Spn density, including the CD8^dim^ T cell cluster (*r* = –0.51, *P* = 0.023) and one of the CD161^+^CD8^+^ T cell clusters (*r* = –0.51, *P* = 0.021) that was increased in carriage^–^ subjects over carriage^+^ subjects. The other 2 clusters that negatively associated with density were also were CD8^+^ T cell clusters, with 1 expressing CD161. On day 7, only 2 clusters were significantly associated with Spn density: the CD8^dim^ T cell cluster (*r* = –0.55, *P* = 0.011) and 1 B cell cluster (*r* = –0.45, *P* = 0.049).

### Nasal B cells are depleted during pneumococcal carriage.

**Figure 3:**
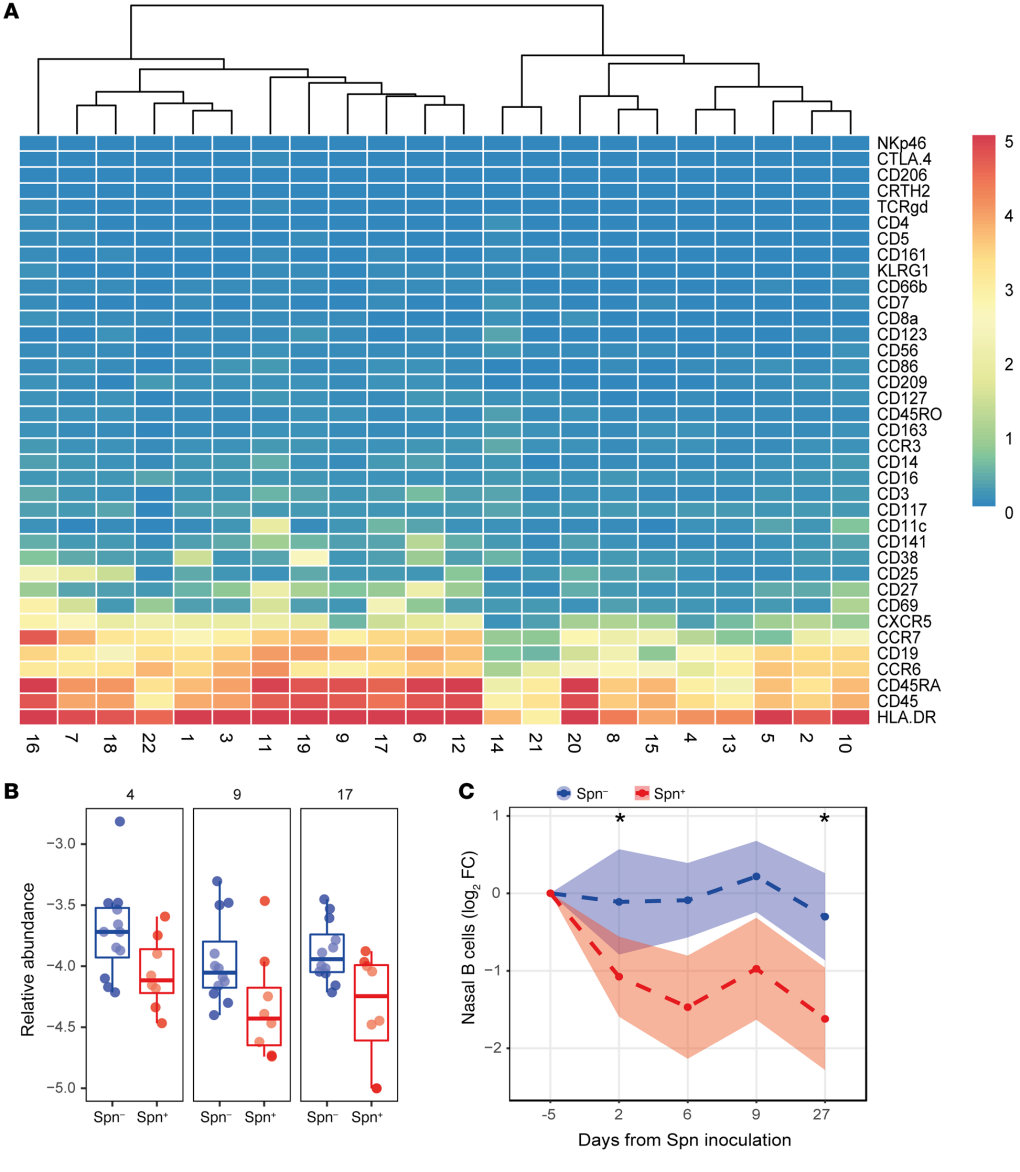
Nasal B cells are depleted following pneumococcal carriage. (**A**) Heatmap showing the expression of 37 markers for all B cell clusters. Clusters were ordered based on similarity, and a distance dendrogram is depicted. (**B**) The relative abundance for each of the 3 significantly higher clusters normalized to stromal cells is expressed on a log_10_ scale for carriage^–^ (Spn–, blue, *n* = 12) and carriage^+^ (Spn+, red, *n* = 8) subjects. Box plots, depicting median and interquartile ranges, with whiskers extending to 1.5× interquartile range or maximum value, and individual subjects are shown. (**C**) Levels of CD19^+^ nasal B cells longitudinally measured by flow cytometry from minimally invasive nasal curettes in an independent cohort for carriage^–^ (Spn–, blue, *n* = 52) and carriage^+^ (Spn+, red, *n* = 42) subjects. Mean and standard error of the mean of log_2_-transformed fold-change levels relative to baseline are shown. *P < 0.05 by Wilcoxon’s test compared with baseline with Bonferroni’s correction for comparing multiple time points.

We then further investigated the 3 B cell clusters that were higher in carriage^–^ subjects (Figure 3, A and B). All 3 significantly higher clusters (cluster 4, 9, and 17) expressed CD45RA, HLA-DR, CD19, CCR6, and CCR7 to varying degrees. None of these clusters expressed CD38, a marker for plasmablasts, or CD5, a marker for innate B cells (38, 39). Cluster 9 was 2.9-fold higher in carriage^–^ subjects (*P* = 0.047) and cells in this cluster expressed also low levels of CXCR5 and CD27. Cluster 17 (2.0-fold higher, *P* = 0.049) additionally expressed the B cell activation marker CD69. To assess whether the higher frequency in carriage^–^ subjects was related to increased B cells in carriage^–^ subjects or decreased B cells in carriage^+^ subjects, we longitudinally measured CD19^+^ B cell frequencies in nasal microsamples collected from an independent cohort (Figure 3C and Supplemental Figure 1A). Compared with baseline, B cell levels decreased following pneumococcal carriage on days 2 (2.1-fold, *P* = 0.048), 6 (2.8-fold), 9 (2.0-fold), and 27 (3.1-fold, *P* = 0.028) after inoculation. In the carriage^–^ group, B cell levels decreased 1.1-fold on days 2 and 6, increased 1.2-fold on day 9, and decreased 1.2-fold on day 27 and were thus relatively stable. The fold-change decrease in nasal B cell levels did not associate with Spn density at any time point.

### Pneumococcal carriage increases circulating plasmablasts.

**Figure 4:**
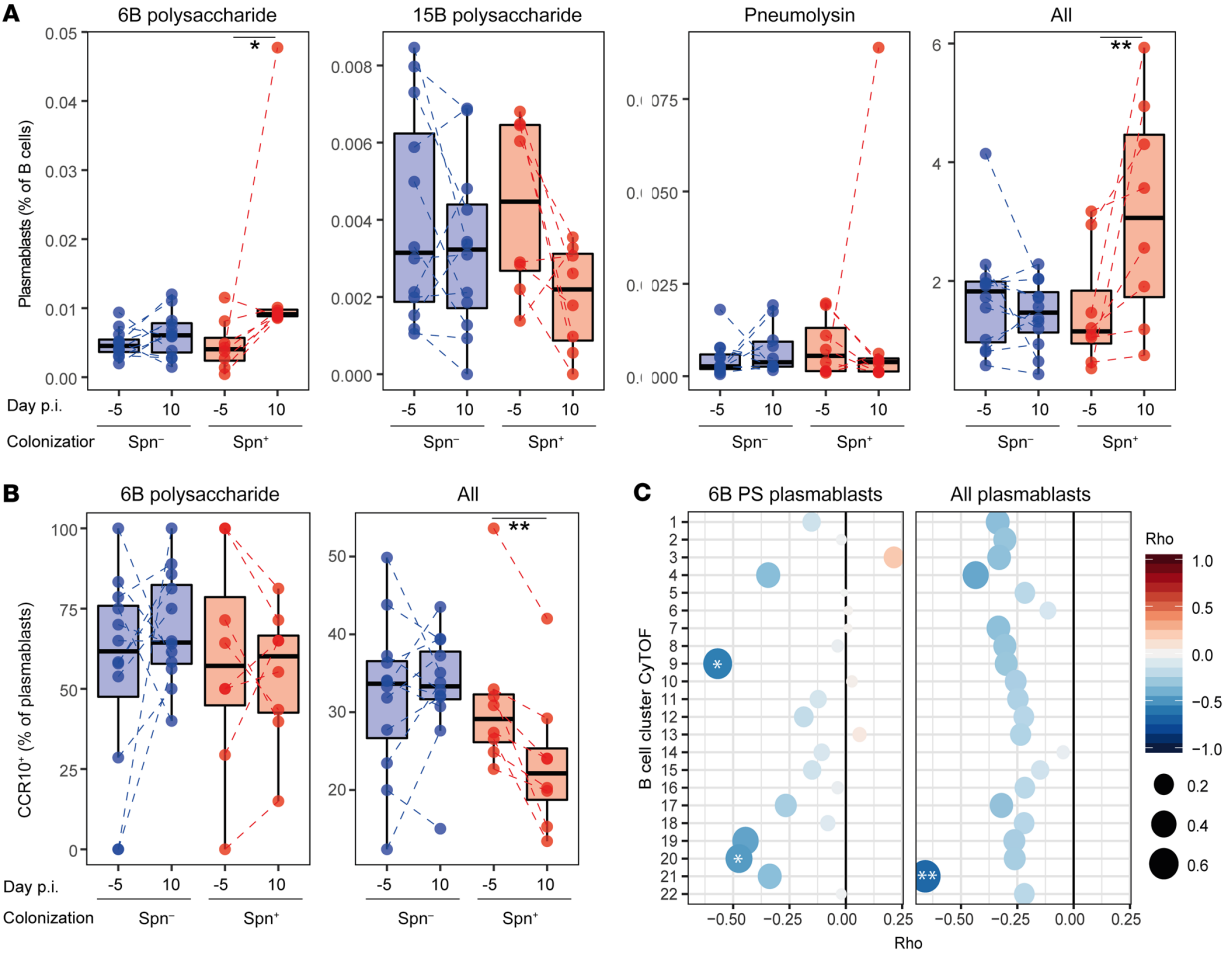
Pneumococcal carriage leads to increased systemic plasmablasts. (**A**) Levels of 6B polysaccharide–specific, 15B polysaccharide–specific, pneumolysin derivative b–specific (Pneumolysin), or all plasmablasts among total B cells were measured from PBMCs collected at baseline (day –5) and at the time of biopsy (day 10 after inoculation). Box plots depicting median and interquartile ranges, with whiskers extending to 1.5× interquartile range or maximum value, and individual subjects are shown with carriage^–^ in blue (*n* = 12) and carriage^+^ in red (*n* = 8). Paired samples are connected by dashed lines. *P < 0.05, **P < 0.01 by Wilcoxon’s test comparing a group to its baseline. (**B**) Levels of CCR10^+^ plasmablasts for 6B-specific and total plasmablasts measured from PBMCs collected at baseline (day –5) and at the time of biopsy (day 10 after inoculation [p.i.]). Box plots and individual subjects are depicted with carriage^–^ in blue and carriage^+^ in red, with paired samples connected by dashed lines. **P < 0.01 by Wilcoxon’s test comparing a group to its baseline. (**C**) Correlations between fold-change in levels of 6B polysaccharide–specific and total plasmablasts between baseline and day 10 against levels of B cell clusters measured by CyTOF. PS, polysaccharide. Color and size of symbols reflect Spearman’s rho value. *P < 0.05, **P < 0.01 by Spearman’s test.

We hypothesized that the depletion of B cells from the nasal mucosa following carriage establishment was due to a recirculation of activated B cells. Although none of the B cell clusters that were lower in the carriage^+^ group expressed the plasmablast marker CD38, it has been demonstrated previously that memory B cells differentiate rapidly into plasmablasts upon activation (40). Therefore, we measured numbers of Spn-specific and total plasmablasts in peripheral blood mononuclear cells (PBMCs) collected before and after carriage establishment using a flow cytometry–based assay (Supplemental Figure 1B). During carriage, the frequency of 6B polysaccharide–specific plasmablasts among total B cells increased, while the frequency of plasmablasts specific for the pneumococcal protein pneumolysin remained unaltered (Figure 4A). As a negative control we measured levels of plasmablasts specific for an unrelated Spn capsular type (15B), which were not affected, as expected. However, the frequency of total circulating plasmablasts among all B cells increased (median 1.5-fold, IQR: 1.2- to 2.8-fold; *P* = 0.008), suggesting that nasal B cells became nonspecifically activated during carriage. Similar results were obtained when normalizing to the total number of lymphocytes, demonstrating that this was not due to other shifting B cell populations (Supplemental Figure 2A). We then investigated CCR10 expression on these plasmablasts, which has been reported to mark IgA-secreting cells (41) and is potentially important for homing of B cells to mucosal tissues including the airways (42, 43). The total population of plasmablasts after carriage displayed reduced numbers of CCR10^+^ cells, in contrast with 6B-specific plasmablasts, indicating differential expansion between specific and nonspecific B cell populations (Figure 4B). This is supported by the observation that increased circulating levels of 6B polysaccharide–specific plasmablasts inversely correlated with the nasal B cell CyTOF clusters 9 and 20, while total plasmablast increases inversely correlated with the CyTOF B cell clusters 21 (Figure 4C). Clusters 9 and 21 still negatively correlated with levels of circulating 6B-specific and total plasmablasts, respectively, after normalization to total lymphocyte numbers (Supplemental Figure 2B). Thus, we wanted to assess whether both Spn-specific as well as unrelated B cells became activated following carriage, leading to recirculation. We therefore measured antibody levels in serum against not only Spn but also *Streptococcus pyogenes*, *Staphylococcus aureus*, and *Haemophilus influenzae* (Hi), as these are common colonizers of the human nasopharynx and thus nasal B cells against these bacterial species are likely present in the nose of most individuals. Following Spn colonization, IgG levels specific for Spn (median 1.4-fold, IQR: 1.1- to 2.4-fold) and Hi (median 1.2-fold, IQR: 1.1- to 1.5-fold) significantly increased, while IgG levels specific for *Streptococcus pyogenes* and *Staphylococcus aureus* were not significantly altered (Supplemental Figure 3A). Serum IgA concentration only increased for Spn and not for Hi or any of the other bacterial species (Supplemental Figure 3B). To investigate whether this observed increase in Hi-specific IgG was due to an increase in cross-reactive antibodies, directed against for example the surface antigen choline phosphate (ChoP) or the capsular polysaccharide (44, 45), we also measured Hi-specific IgG titers following adsorption with whole-cell pneumococcus (Supplemental Figure 3C). This abrogated the increased titers against Hi after carriage, indicating this was likely due to cross-reactivity. Similarly, in nasal wash, levels of IgA against Spn and Hi were increased, whereby the latter also was prevented by preadsorption of the nasal wash with Spn (Supplemental Figure 3, D and E).

### Nasal CD8^+^ tissue-resident memory T cells are higher in carriage^–^ subjects.

**Figure 5:**
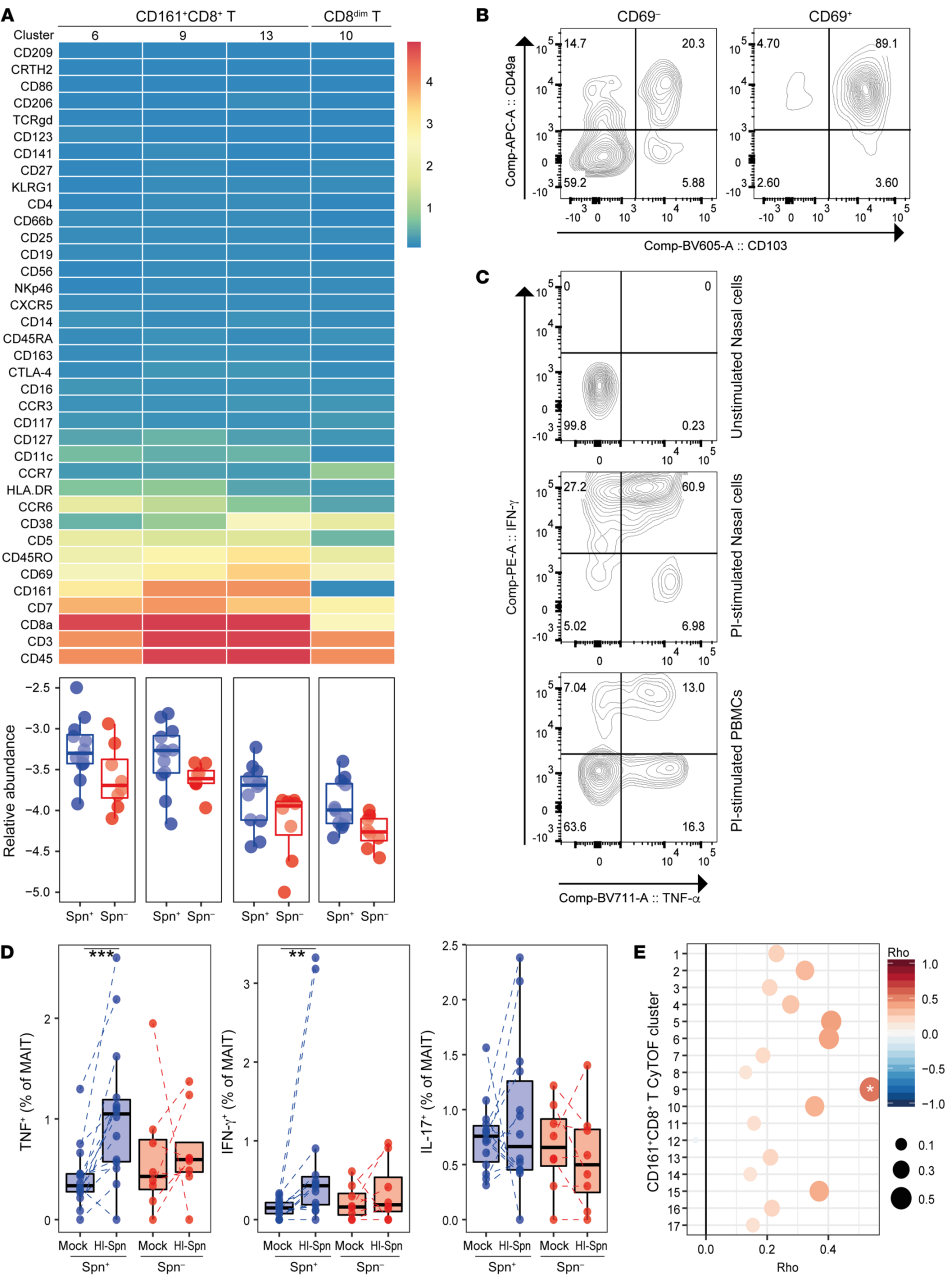
Increased MAIT cell responses associate with protection from carriage. (**A**) Heatmap showing the expression of 37 markers for each of the 4 CD8^+^ clusters that were significantly different between carriers and noncarriers. Nonsignificant CD8^+^ T clusters are not shown. Below the heatmap, the abundance for each of the significantly higher clusters normalized to stromal cells is expressed on a log_10_ scale for carriage^–^ (blue) and carriage^+^ (red) subjects. Box plots depicting median and interquartile ranges, with whiskers extending to 1.5× interquartile range or maximum value, and individual subjects are depicted. (**B**) Representative flow cytometry contour plot of CD69^+^CD8^+^ and CD69^–^CD8^+^ T cells, showing CD103 and CD49a tissue-resident marker expression on nasal biopsy cells (*n* = 4). (**C**) Representative flow cytometry contour plot of unstimulated nasal biopsy cells, and nasal biopsy cells and PBMCs stimulated overnight with PMA and ionomycin (PI) to assess functional capacity (*n* = 4). (**D**) TNF-α, IFN-γ, and IL-17A production by CD8^+^ MAIT cells (CD161^+^TCRvα7.2^+^) after 3-day in vitro stimulation with heat-inactivated pneumococcus (HI-Spn) or left unstimulated for carriage^–^ (blue, *n* = 14) and carriage^+^ (red, *n* = 8) subjects in PBMCs collected at baseline. Box plots and individual subjects, connected by dashed lines, are shown. **P < 0.01, ***P < 0.001 by Wilcoxon’s test. (**E**) Correlations between the difference in cytokine production (total of TNF-α and IFN-γ) by MAIT cells in vitro stimulated with HI-Spn or left unstimulated against CD161^+^CD8^+^ T cell clusters measured by CyTOF (*n* = 20). Color and size of symbols reflect Spearman’s rho value. *P < 0.05 by Spearman’s test.

The 3 clusters of CD8^+^ T cells and the cluster of CD8^dim^ T cells that were higher in carriage^–^ subjects all expressed CD69, a marker of tissue-resident memory T (Trm) cells (Figure 5A). To verify that these CD69^+^CD8^+^ T cells represented Trm cells, we measured the expression of CD103 and CD49a on CD69^+^ and CD69^–^ cells by flow cytometry from a representative biopsy (Supplemental Figure 4A). Indeed, 89.1% of nasal CD69^+^CD8^+^ T cells expressed CD103 and CD49a, confirming that these were Trm cells (Figure 5B) (46). The markers CD5, CD38, HLA-DR, CCR6, CD127, CCR7, and CD11c were expressed in cluster-specific patterns and at varying intensities among the significant clusters. This suggests that clusters of cells with varying degrees of activation and memory types were enriched in carriage^–^ subjects. One cluster expressed only low levels of CD8 (cluster 10 of CD8^dim^ T cells, 2.0-fold higher, *P* = 0.016), which could reflect cytotoxic effector memory cells (47). We then stimulated nasal biopsy cells and PBMCs overnight with PMA and ionomycin to assess the functional capacity of nasal CD8^+^ T cells (Figure 5C). Among nasal CD8^+^ T cells, 94.8% produced tumor necrosis factor α (TNF-α) and/or IFN-γ following stimulation, compared with 36% of blood CD8^+^ T cells, demonstrating that nasal CD8^+^ T cells are highly functional.

### Baseline circulating MAIT functionality associates with resistance to pneumococcal carriage.

Three of the 4 significant clusters expressed CD161, a marker for mucosa-associated invariant T (MAIT) cells, and we therefore tested the hypothesis that MAIT cell responses against Spn were associated with protection against carriage. PBMCs collected prior to pneumococcal challenge were stimulated in vitro with heat-inactivated Spn and activation (CD69) and cytokine production (TNF-α, IFN-γ, and IL-17A) were assessed (Supplemental Figure 4B). MAIT cells of both carriage^–^ and carriage^+^ groups upregulated CD69 after a 3-day culture with heat-inactivated Spn (Supplemental Figure 4C). However, only MAIT cells from carriage^–^ subjects produced increased levels of TNF-α and IFN-γ, but not IL-17A, upon restimulation in vitro with heat-inactivated Spn (Figure 5D). Conversely, MAIT cells from carriage^+^ subjects did not produce increased levels of any cytokine upon stimulation. This was specific to MAIT cells, as conventional CD8^+^ T cells responded by producing small amounts of IFN-γ and no TNF-α (Supplemental Figure 4D). The baseline responses of MAIT cells in blood upon restimulation showed a positive correlation with numbers of nasal cells 10 days after pneumococcal challenge in CyTOF CD161^+^CD8^+^ T cell cluster 9, which was significantly higher in the carriage^–^ group (*r* = 0.54, *P* = 0.02, Figure 5E).

To assess the kinetics of nasal MAIT cells, we collected nasal curettes before and at 2 and 6 days after inoculation in an independent cohort and analyzed total MAIT cells by flow cytometry (Supplemental Figure 5). Total MAIT cell numbers were similar between carriage^–^ and carriage^+^ groups at all time points, as observed by CyTOF on day 10. Due to the low numbers of cells that can be obtained using minimally invasive curettage, we were not able to longitudinally measure MAIT cell subsets that were significantly associated with carriage as identified by CyTOF.

### Association between baseline IgG and abundance of B and CD8^+^ T cell clusters.

**Figure 6:**
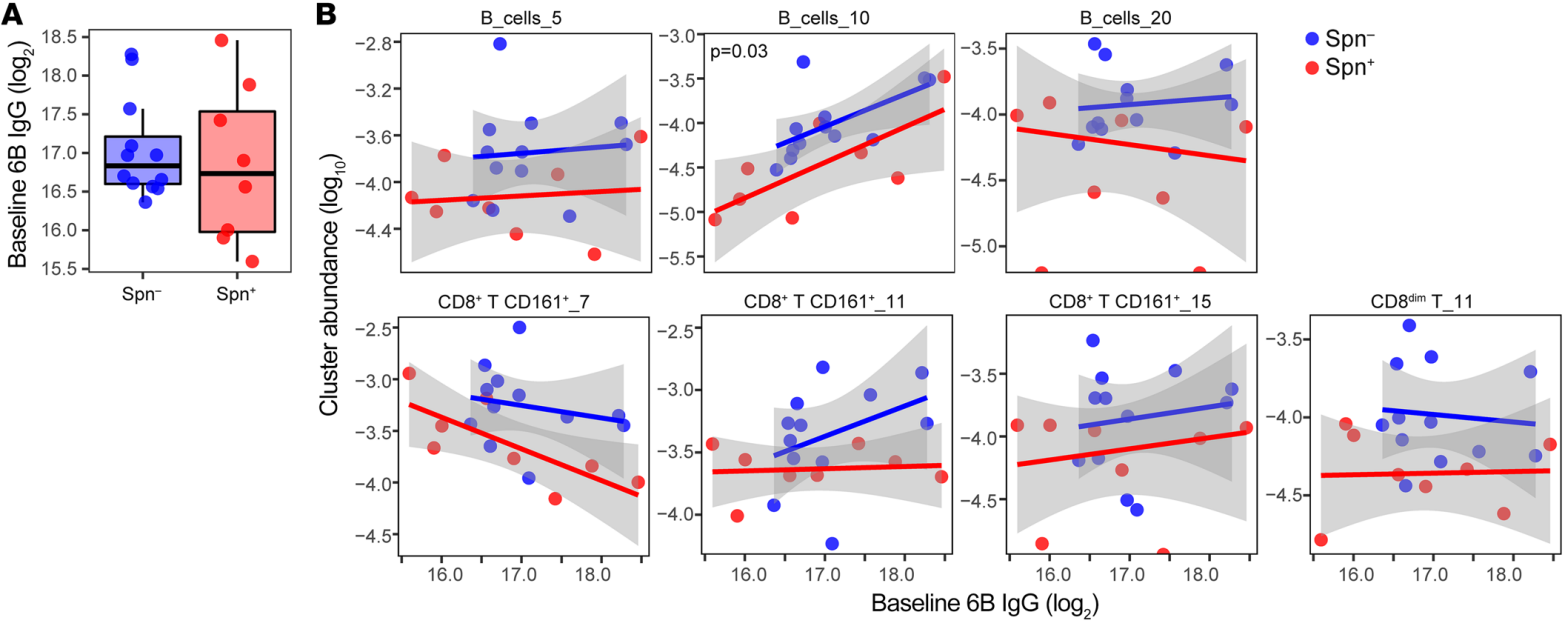
Association between baseline IgG against Spn and cluster abundance. (**A**) Levels of baseline IgG levels against whole-cell 6B Spn in serum are shown for carriage^–^ (blue, *n* = 12) and carriage^+^ (red, *n* = 8) subjects. Box plots depicting median and interquartile ranges, with whiskers extending to 1.5× interquartile range or maximum value, and individual subjects, connected by dashed lines, are shown. (**B**) Correlation between abundance of clusters and baseline levels of IgG are shown. Individuals subjects and regression lines per group are shown, with 95% confidence intervals depicted in gray. *P = 0.03 determined by linear regression model including carriage status and log-transformed baseline IgG levels.

We wanted to further characterize the relationship between abundance of the clusters that were significantly different between carriage^+^ and carriage^–^ groups with levels of baseline IgG against Spn to assess whether recall responses were involved. As previously reported, there was no difference between carriage^+^ and carriage^–^ groups in levels of baseline IgG against Spn (Figure 6A) (7, 25, 27). Of the 7 clusters significantly associated with carriage status, 1 cluster (B cell cluster 9) showed a positive association between baseline IgG and cluster abundance (Figure 6B). This B cell cluster also correlated with the increased number of 6B-specific plasmablasts following colonization, which could suggest that these B cells are linked to production of Spn 6B–specific antibodies.

### Nasal monocytes show limited differentiation into macrophages.

Monocytes have been previously associated with the clearance of Spn carriage (16, 23); however, these cells have not been previously phenotyped in detail in the human nasopharynx. Of the 25 clusters defined in the myeloid lineage, 15 expressed CD14 (Supplemental Figure 6). Of these, only 2 also expressed CD16. Four CD14^+^ clusters expressed the macrophage markers CD163 and CD206 and an additional 3 clusters expressed CD206 but not CD163 (48). However, alveolar monocytes can express CD206, suggesting this is not a definitive indication of differentiation (49). The activation markers CD25 and CD86 were present on 5 monocyte clusters (50). Thus, monocytes/macrophages in the nose mainly consisted of classical monocytes with limited differentiation into macrophages.

### Characterization of nasal CD4^+^ memory T cells.

CD4^+^ T memory cells, in particular Th17 cells, were previously found to be critical for Spn immunity in mouse models of nasal colonization (15, 16). Of all cells in the CD4^+^ T cell lineage, 89.6% expressed the memory marker CD45RO. Of these, 60.3% expressed CD161, a marker that has been proposed to identify Th17 cells (51, 52). Another 4.6% of memory cells were defined by expression of high levels of CD25, a marker for regulatory T cells. We defined 23 clusters of CD161^–^CD4^+^ T memory cells, 21 clusters of CD161^+^CD4^+^ T memory cells, and 9 clusters of CD25^hi^CD4^+^ T memory cells (Supplemental Figure 7). All CD4^+^ T memory cell clusters expressed a combination of the markers CD7, CD127, HLA-DR, CD38, and CD69, demonstrating a wide range in activation and differentiation status (46). The CD25^hi^CD4^+^ T memory cells likely were regulatory T cells, as they were predominantly negative for CD127 and 2 of these clusters expressed cytotoxic T lymphocyte–associated protein 4 (CTLA-4) and CD27 (53). CD161 was not restricted to Th17 cells, as among CD161^+^CD4^+^ T memory cells, 2 clusters also expressed CD8 and were thus double-positive T cells (54). In addition, 2 clusters expressed CD25 without CD127 expression, indicating regulatory T cells, and 1 cluster expressed chemoattractant receptor–homologous molecule expressed on Th2 cells (CRTH2), a marker of Th2 cells (55).

### Cellular distribution through the nasal mucosa.

**Figure 7:**
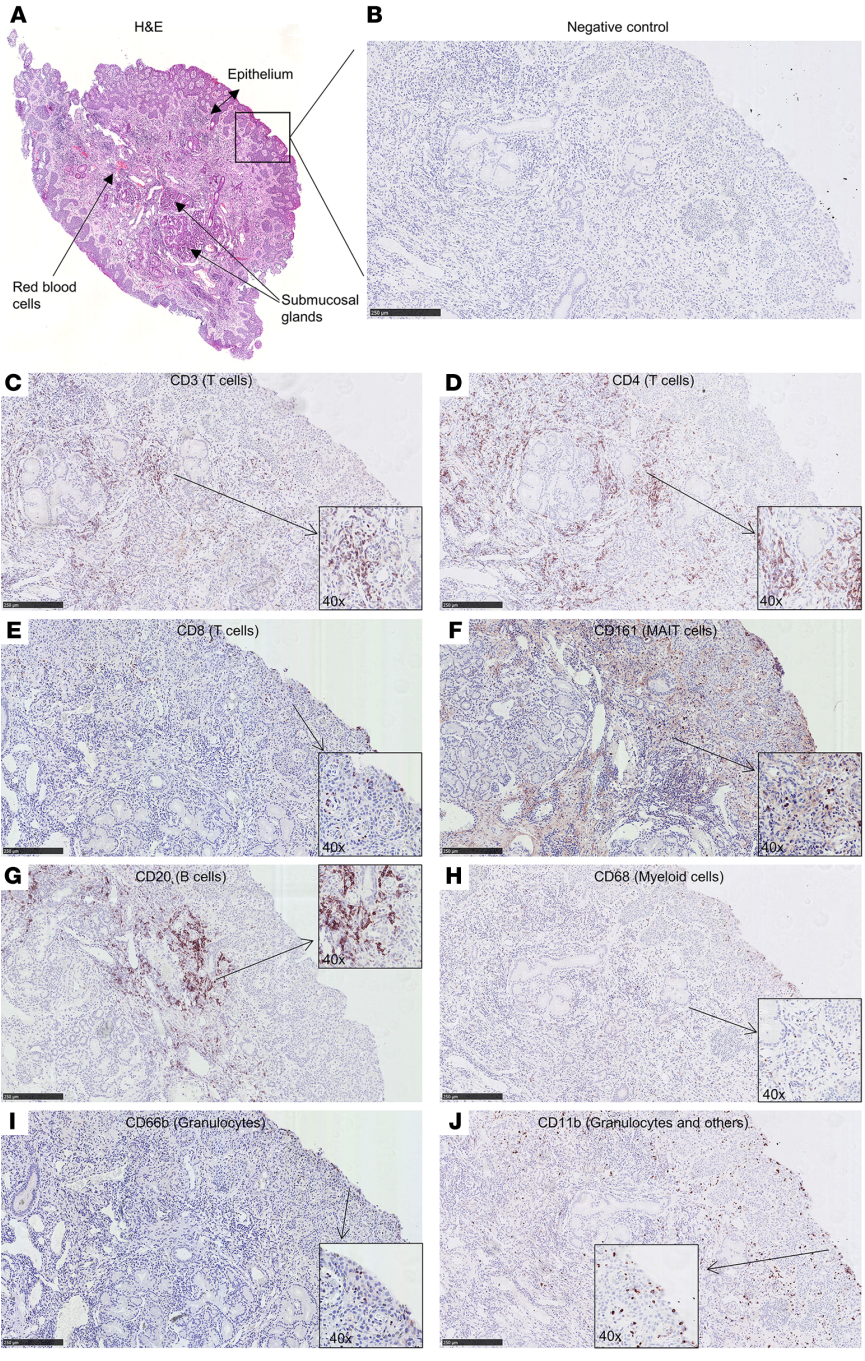
Immunohistochemistry on serial sections of a nasal biopsy. To establish an overall cellular distribution in the tissue, an ×10 magnification is shown for each of the markers. Insets (×40 magnification) are also included to visualize some individual positive cells. (**A**) Hematoxylin and eosin (H&E) staining showing the entire biopsy. Staining of subsequent slices showing the biopsy at the epithelial edge for the markers (**B**) negative control, (**C**) CD3, (**D**) CD4, (**E**) CD8, (**F**) CD161, (**G**) CD20, (**H**) CD68, (**I**) CD66b, and (**J**) CD11b. Scale bars: 250 μm. Slices were counterstained with H&E. Some background staining of the extracellular matrix is present for CD161 (**F**). Biopsy was derived from 1 challenged, carriage^–^ subject.

We then performed immunohistochemistry on a biopsy from a challenged but carriage^–^ subject to further understand the distribution of these cells through the mucosal tissue (Figure 7). CD4^+^ T cells were found predominantly in the subepithelial layer (Figure 7, C and D), while CD8^+^ and CD161^+^ cells were also found at the epithelial layer (Figure 7, E and F). Similarly to CD4^+^ T cells, B cells (defined by CD20) were mostly observed in the subepithelium, while myeloid cells (CD68) could be seen at both the epithelial and subepithelial layer (Figure 7, G and H). Neutrophils were found abundantly at the epithelial surface but also in the subepithelium (Figure 7, I and J).

## Discussion

This study comprehensively characterized immune cells in biopsies collected from the human nasal mucosa. As nasal samples were collected 10 days following experimental human pneumococcal challenge, we were able to associate the frequency of specific immune populations with Spn carriage. Given the difficulty in access to such tissue samples, especially in a setting where the onset of infection is known, this provided a unique opportunity to investigate mucosal immune responses not undertaken previously. The application of CyTOF led to a broad and comprehensive study of cellular subsets involved in immunity against Spn carriage, deriving 293 immune clusters belonging to 9 cellular lineages. Clusters belonging to B cells and CD161^+^CD8^+^ T cells were higher in carriage^–^ subjects. In addition to carriage status, we also associated Spn density with cluster abundance. Several CD8^+^ T cell clusters negatively correlated with Spn load, further supporting a protective function for these cells.

B cells were depleted from the nasal mucosa following the establishment of Spn carriage. This depletion correlated on an individual level with increased numbers of circulating 6B polysaccharide–specific and total plasmablasts. Thus, this depletion likely was due to recirculation of activated B cells rather than due to apoptosis of nasal B cells upon Spn polysaccharide capsule encounter, as has also been described (56). The total plasmablast expansion, but not 6B plasmablast expansion, was characterized by a decreased proportion of CCR10^+^ cells, suggesting a preferential expansion of CCR10^–^ cells or a downregulation of this marker. The correlation between low numbers of cells in specific nasal B cell clusters with increased levels of circulating plasmablasts indicates that activation of nasal B cells during carriage led to B cell recirculation. In particular, numbers of B cell cluster 9, which were lower in carriage^+^ subjects, associated with 6B polysaccharide–specific plasmablasts, as well as with levels of pneumococcus-specific antibodies in serum. Indeed, trafficking of memory B cells between airways and blood has been reported (57).

Levels of serum IgG against Hi increased following colonization with Spn, due to an induction of cross-reactive antibodies, as preadsorption with Spn abrogated this increase. Thus, pneumococcal colonization has an effect on mucosal and systemic B cell populations and antibodies that bind both pneumococcus and Hi. The negative association between Spn and Hi in the human nasopharynx is well described and this observation could be added to the potential mechanisms that underlie this interaction, such as Hi-mediated recruitment of neutrophils and clearance of pneumococcus (58). B cells express the innate receptors TLR2 and TLR4 (59), which can be activated by pneumococcus, and we thus hypothesized that pneumococcal carriage leads to nonspecific activation of B cells. *Neisseria lactamica* has been previously demonstrated to be able to nonspecifically activate innate B cells (60, 61). However, the increased antibody responses against Hi were likely due to induction of cross-reactive antibodies and we observed no increase in serum IgG levels against *Streptococcus pyogenes* or *Staphylococcus aureus*, 2 other common nasal colonizers.

Several nasal CD8^+^ Trm cell clusters were higher in subjects protected from Spn carriage. These cells were previously found to be protective against influenza infection in murine models (62). Spn is classically thought of as an extracellular bacterium and therefore the role of CD8^+^ T cells in controlling Spn has not been extensively studied in humans. However, it was recently shown that Spn can replicate within splenic macrophages and can reside within epithelial cells, suggesting that CD8^+^ T cell immunity could be elicited by Spn and play a role in protection against Spn carriage or disease (63, 64). Indeed, Spn protein–specific CD8^+^ T cells could be readily detected in blood of Gambian adults (65). In murine models, CD8^+^ T cells were found to be protective against Spn lung infection but did not have an effect on nasopharyngeal carriage (19, 66).

We found here that CD8^+^ MAIT cell functionality before pneumococcal challenge associated with a resistance to carriage acquisition. MAIT cells were recently reported to be able to recognize Spn through MHC class I–related protein 1–dependent (MR-1–dependent) and –independent pathways (67). MAIT cells were previously found to be important in the protection against lung bacterial and viral infections via direct and indirect responses (68). Our findings now suggest these cells could also protect against nasopharyngeal Spn colonization. Given the abundance of MAIT cells at the nasal mucosa and their specificity for precursors from the riboflavin synthesis pathway, which is highly conserved in the Spn genome (67), these cells are excellently placed to initiate an immune response upon exposure to Spn. The rapid production of cytokines such as TNF-α and IFN-γ by these cells upon Spn encounter could lead to the recruitment or activation of neutrophils and monocytes, which in turn could phagocytose Spn and protect against carriage acquisition (69). Baseline MAIT functionality in blood positively correlated with cell numbers within one of the nasal CD161^+^CD8^+^ cell clusters, suggesting trafficking of MAIT cells from the blood to the nose upon pneumococcal encounter. Indeed, MAIT cells have been shown to be depleted from the circulation and accumulate in tissues upon infection (70, 71).

One limitation of this study is that the number of granulocytes measured was very low due to the overnight resting step following enzymatic digestion. While this resting step allowed for the return of markers that were cleaved by the enzymatic digestion, neutrophils quickly become apoptotic after being removed from the body (72–74). Consequently, the characterization of granulocytes reported here is incomplete and we were not able to assess whether specific neutrophil subsets are associated with protection against pneumococcal colonization.

In addition, due to the invasiveness of sample acquisition, sample size was limited and we were not able to characterize nasal biopsies at various time points. Thus, no baseline was available, making it impossible to conclude whether differences between carriage^–^ and carriage^+^ groups were present at baseline or occurred in the 10 days following inoculation. In addition, we were not able to assess transient responses early after bacterial inoculation. To address this caveat, we longitudinally measured levels of nasal B cells and MAIT cells collected by nasal curettes in independent cohorts. This analysis demonstrated that B cells were depleted from the nose upon colonization. In contrast, we did not observe any changes in total numbers of MAIT cells. Using CyTOF, we observed that MAIT cell clusters expressing the markers CD7, CD69, CD5, and CCR6 or CD38, but not total number of MAIT cells, were higher in the carriage^–^ group. Thus, the larger amount of cells obtained from nasal biopsies combined with the broad analysis by CyTOF allowed us to identify subpopulations of MAIT cells associated with protection against colonization.

This study revealed some notable differences from previously conducted experiments with murine models. In particular, we did not see any association between Th17 cells, or any CD4^+^ T memory cells, and control of colonization as previously reported (15, 16). This agrees with previous observations from experimentally colonized adults that nasal IL-17A levels are not increased (24). Moreover, there was no association between monocytes and Spn colonization status or density, unlike what was previously reported in murine models or humans followed up longitudinally following experimental colonization (16, 20, 24). This is possibly because we terminated carriage after day 7 for safety reasons prior to collecting biopsies, making it impossible to associate immune cell clusters, such as monocytes, with Spn clearance, which typically occurs after day 10. Moreover, due to relatively small numbers of individuals in this controlled human infection study, it is possible that subtle associations between cell populations and bacterial load were missed. This study, however, does provide a unique characterization of monocyte/macrophage phenotypes in the human nose, which show remarkably little differentiation into macrophages. Another remarkable difference between this study and findings from murine models is that we observed a protective effect of MAIT cells against colonization, while depletion of CD8^+^ T cells did not affect immunological Spn control in mouse models (19). MAIT cells are a recently identified T cell subset that is common in humans, consisting of up to 10% of all T cells in the circulation, but that is very rare in mice (68). It is possible that this difference has led to an underappreciation of the CD8^+^ T cell’s role in protection against pneumococcal carriage in humans. Finally, our finding of an activation and exodus of B cells from the nose merits further attention and validation using tractable mouse models to understand its role in the generation of humoral immunity against Spn and cross-reactive protection against Hi.

In conclusion, this study provides both a broad and in-depth view of the adult human nasal immune system in the setting of experimental human pneumococcal challenge. Nasal B cells were depleted following carriage establishment, likely due to differentiation to plasmablasts and recirculation. In addition, CD8^+^ MAIT cell responses were associated with protection from Spn carriage.

## Methods

### Study design and sample collection.

Healthy adult subjects were screened for the presence of natural pneumococcal carriage in nasal wash (NW) samples using classical microbiology (7, 34, 75). Subjects not naturally carrying pneumococcus were then inoculated with 80,000 CFU per nostril of 6B type Spn as described previously (7, 75). Development of nasal carriage was monitored using NW samples collected on days 2 and 7 after inoculation. Growth of pneumococcus from NW samples at any time point defined carriage^+^ volunteers. All subjects then received a 3-day course of amoxicillin and underwent a 4-mm nasal biopsy on day 10 after inoculation. The nasal cavity was first sprayed up to 6 times with 5% lidocaine hydrochloride with 0.5% phenylephrine hydrochloride. Five to 10 minutes later, the infero-medial part of the inferior turbinate, i.e., the point of incision, was injected with up to 1 mL of 2% lidocaine hydrochloride with adrenaline (1:80,000). An incision of approximately 5 mm with a No. 15 blade was then made and 2–4 mm of mucosal tissue was removed with Tillies Henckle’s surgical forceps. This study was registered under ISRCTN85509051. Nasal curettes (ASL Rhino-Pro, Arlington Scientific) were collected from an additional cohort (ISRCTN16993271) of subjects inoculated with the same 6B strain. The outcomes reported in this manuscript were a priori included in the study protocols.

### Nasal biopsy digestion.

Nasal biopsies were finely cut using a sterile scalpel size 11 (Thermo Fisher Scientific). Pieces were then incubated in 20 mL prewarmed RPMI 1640 (Thermo Fisher Scientific) with Liberase TL (250 μg/mL, MilliporeSigma) and DNAse I (50 μg/mL, MilliporeSigma). Fragments were incubated for 45 minutes at 37°C, while shaking at 250 rpm at a 10° angle. At the end of the digestion, biopsies were passed 5 times through a 16-gauge blunt-ended needle (Thermo Fisher Scientific) and the digested sample was filtered over a 70-μm filter (Thermo Fisher Scientific). This process was repeated for any remaining fragments. Cell were spun down for 10 minutes at 400 *g* and then red blood cells were lysed using an osmotic lysis buffer. Cells were washed with RPMI with 20% heat-inactivated fetal bovine serum (FBS, Thermo Fisher Scientific), resuspended at 1 × 10^6^ cells/mL in RPMI with 20% FBS, and rested overnight. The next day, cells were counted and washed with RPMI containing 10% FBS. Cells were stained using 1 μM intercalator Rh-103 (Fluidigm) as a viability marker for 15 minutes, washed, and fixed with 1.8% paraformaldehyde (PFA) (MilliporeSigma) for 15 minutes. Cells were washed and stored in liquid nitrogen in CTL-Cryo ABC media (Cellular Technology Limited) until CyTOF barcoding and staining.

### Mass cytometry staining and analysis.

Nasal biopsy cells were thawed on ice and barcoded using the Cell-ID 20-plex Pd Barcoding Kit as per the manufacturer’s instructions (Fluidigm). The effect of fixation on epitopes detected by the included antibody clones was tested using PBMCs and monocyte-derived dendritic cells. Following 3 washes with staining buffer (Fluidigm) and 10 minutes of FcR blocking (BioLegend), pooled cells were stained for 45 minutes at room temperature with the antibody cocktail (Supplemental Table 1). All Fluidigm antibodies were preconjugated to metals, while all other antibodies were conjugated using a total of 100 μg of purified antibody combined with the MaxPar ×8 Antibody Labeling Kit (Fluidigm) according to manufacturer’s protocol v.7 and stored in 200 μL Antibody Stabilizer PBS (Candor Bioscience) at 4°C. Cells were washed twice with staining buffer and incubated for 1 hour with 1:1,000-diluted 125 μM Cell-ID intercalator-Ir (Fluidigm) to stain DNA. Cells were washed 3 times with staining buffer and 2 times with deionized H_2_O prior to addition of normalization beads (Fluidigm) and acquisition on a Helios 2 mass cytometer (DVS Sciences). CyTOF Fcs files were normalized using the included beads, concatenated, and debarcoded as per the manufacturer’s instructions. The debarcoding step leads to a removal of doublets (76). Then, viable immune cells were pregrated (Figure 2) and exported as .fcs files using FlowJo X (Tree Star). These were further analyzed using Cytosplore (https://www.cytosplore.org/).

### Nasal B and MAIT cell phenotyping.

Immunophenotyping of nasal B and MAIT cells obtained by curettes was performed as described previously (29). In brief, cells were dislodged from curettes and stained with LIVE/DEAD Fixable Aqua Dead Cell Stain (Thermo Fisher Scientific) and an antibody cocktail containing, among others, Epcam-PE, HLADR-PE/Cy7, CD66b-FITC, CD19-BV650 (all BioLegend), CD3-APC/Cy7, CD14-Percp/Cy5.5 (BD Biosciences), and CD45-PACOrange (Thermo Fisher Scientific) for B cells, while the cocktail for MAIT cells also included CD8–BV785 and TCRva7.2-BV711 or TCRva7.2–PE/Texas Red and CD45-BV510 (BioLegend). Samples were acquired on a BD LSR II flow cytometer and analyzed using FlowJo X. Fluorescence-minus-one controls for each of the included antibodies were used to validate results during setup of all of the panels used. Samples with less than 500 immune cells or 250 epithelial cells (11.9% of all nasal samples) were excluded from further analysis. A full list of all antibodies used for flow cytometry is provided in Supplemental Table 2).

### Intracellular cytokine staining following PMA/ionomycin or pneumococcus stimulation.

For intracellular cytokine staining after PMA and ionomycin stimulation, fresh nasal biopsy cells or PBMCs were stimulated with 100 and 500 ng/mL of these, respectively. After 2 hours, GolgiPlug (BD Biosciences) was added and cells were incubated for another 16 hours. Cells were washed and stained extracellularly with LIVE/DEAD Fixable Violet Dead Cell Stain for 15 minutes and then for another 15 minutes with CD161-APC, CD69-BV650, CD25-PEDazzle594, CD103-BV605, CD4-PercpCy5.5, CD8–Alexa Fluor 700, TCRvα7.2-BV785 (all BioLegend); and CD3-APH7 and TCRgd-PECy7 (BD Biosciences). Cells were then permeabilized using the eBioscience Foxp3 Transcription Factor Staining Buffer Set (Thermo Fisher Scientific) following the manufacturer’s protocol. Intracellular staining was done for 30 minutes with FOXP3–Alexa Fluor 488, IFN-γ–PE, TNF-α–BV711 (BioLegend), and IL17A-BV510 (BD Biosciences). Finally, cells were washed, resuspended in 200 μL PBS, and acquired on an LSR II.

For staining with pneumococcus, PBMCs were thawed with 50 μg/mL DNAse I (MilliporeSigma) in prewarmed RPMI containing 10% FBS and washed twice, once in media including DNAse I and once in media without DNAse I. Cells were rested overnight and then cultured at 5 × 10^5^ cells in 500 μL media with 5 μg/mL (corresponding to 4.3 × 10^7^ CFU/mL) heat-inactivated type 6B Spn or left unstimulated as a control. After 48 hours, fresh antigen was added to the cells and 2 hours later GolgiPlug was added and cells were treated as above.

### Pneumococcus-specific B cell detection.

Purified pneumococcal polysaccharides 6B and 15B (Oxford Biosystems) and pneumococcal pneumolysin derivative b (Pdb) were diluted to 100 μg/mL in purified H_2_O and biotinylated using the One-Step Antibody Biotinylation Kit (Miltenyi Biotec) as per the manufacturer’s instructions. Biotinylated proteins were then dialyzed twice for 45 minutes against 1 L of PBS using a Slide-A-Lyzer MINI Dialysis Device (3,500 molecular weight cut off, Thermo Fisher Scientific) and stored at 4°C until labeling. Biotinylated 15B, 6B, and Pdb were then mixed in a 4:1 molecular ratio (Pdb), or a 1:1 molecular ratio (polysaccharides), with PE-streptavidin, BV785-streptavidin, or FITC-streptavidin (BioLegend), respectively. Incubation was performed on ice in a stepwise approach where 1/10 fraction of streptavidin conjugate was added to the antigen followed by a 10-minute incubation. After the final incubation, 1 pmol free biotin was added and the mixture was incubated for 30 minutes on ice. Labeled antigens were stored at 4°C and used within 2 weeks.

To stain cells, PBMCs were thawed with 50 μg/mL DNAse I (MilliporeSigma) in prewarmed RPMI containing 10% FBS and washed once in media including DNAse I. Cells were then resuspended in PBS containing LIVE/DEAD Fixable Violet Dead Cell Stain with 10 μg/mL purified streptavidin (to block nonspecific binding, BioLegend) for 15 minutes. Then, labeled antigens and an antibody cocktail containing CD71–Alexa Fluor 700 (BD Biosciences), CD19-BV605, CD27-PE/Cy7, CD38-APC/Cy7, CD69-BV510, and CCR10-APC (all BioLegend) was added and cells were incubated for another 15 minutes. Finally, cells were washed, resuspended in 200 μL PBS, and acquired on an LSR II.

### Immunohistochemistry.

A nasal biopsy was fixed in 4% PFA for 16–24 hours before rinsing in 50% and 70% ethanol. This was embedded in paraffin, cut into 4-μm sections, dewaxed, subjected to antigen retrieval (95°C for 15 minutes in sodium citrate buffer, pH 6), and processed for immunohistochemistry as described previously (77). In short, sections were permeabilized in methanol for 15 minutes with 1% hydrogen peroxide. After rinsing in PBS, primary antibodies were diluted in goat (or horse) serum buffer (1% BSA, 4% goat [or horse] serum, and 0.01% sodium azide in PBS). Primary antibodies used were CD3 (Dako), CD4, CD20, CD66b, CD68, CD11b (Abcam), CD8 (Epitomics), and CD161 (Atlas Antibodies), which were applied overnight at 4°C (Supplemental Table 3). Sections were rinsed in PBS and secondary biotinylated antibodies (Vector Laboratories) were applied for 45 minutes at room temperature. Slides were rinsed and a complex of avidin and biotin (ABC) solution was added to sections for 60 minutes, which was prepared 30 minutes prior to incubation. After rinsing, NovaRed (Vector) chromogen was prepared according to the manufacturer’s instructions. Sections were counterstained, dehydrated, placed in xylene, and mounted for microscopy and scanned using the NanoZoomer digital pathology (NDP, Hamamatsu Photonics K.K.). Pictures were processed using the NDPview 2 software (version 2.6.13; Hamamatsu Photonics K.K.).

### ELISA.

Serum IgG and IgA titers against Spn*, Streptococcus pyogenes, Staphylococcus aureus*, and Hi were quantified in serum samples, whereas nasal mucosa IgG and IgA titers against Spn and Hi were measured in nasal wash samples using whole-cell ELISA. The ELISA was performed on MaxiSorp 96-well plates (Nunc). Per pathogen, 100 μL of 1 × 10^8^ CFU/mL was prepared in carbonate buffer (pH 8), added to the plates, and allowed to adhere to the wells for 16 hours at 22°C. Then, the plates were washed 3 times using PBS containing 0.05% Tween 20, followed by blocking with an additional 100 μL of PBS containing 2% BSA. Plates were incubated at 37°C for 1 hour and were washed before adding serial dilutions of serum samples. For detection of IgG and IgA, a 1:5,000 and 1:4,000 dilution of anti–human IgG (MilliporeSigma, A9544) and anti–human IgA (MilliporeSigma, A9669), respectively, was made using 0.1% BSA and 100 μL added to each well after washing and incubated at room temperature for 2 hours. Standard curves for IgG and IgA were generated based on a standard serum pool (sera of 7 Spn carriers collected on day 23 after challenge). Arbitrary units of IgG and IgA were assigned to the serum standard for each pathogen. To adsorb antibodies cross-reactive against Spn, pneumococci were added to the samples, followed by a 2-hour incubation at room temperature and overnight incubation on a rotor at 4°C. The next day, samples were centrifuged at 4,000 *g* for 3 minutes and the supernatant was collected and measured as above. Efficacy of depletion was confirmed by ELISA against Spn after adsorption.

### Statistics.

Two-tailed, nonparametric statistical tests were used throughout the study. The number of cells in a cluster for each subject was normalized against the total number of nonimmune cells acquired by CyTOF for that subject to account for number of cells isolated from a given biopsy. This normalization strategy has the advantage that the normalized frequencies of cells in a cluster are not dependent on other clusters, which is a major disadvantage of normalizing against total immune cells. Normalized cluster abundances were then compared between carriage^–^ and carriage^+^ subjects for each of the clusters using the Mann-Whitney test, without correcting for multiple testing. Data were analyzed and graphs were created using ‘pheatmap’ and ‘ggplot2’ packages in R software and a circular graph (Figure 1C) was created using circos software (78). The graphical abstract was created with BioRender.

### Data availability.

Normalized and debarcoded CyTOF .fcs files have been deposited in the FlowRepository with identifier FR-FCM-ZYSE (https://flowrepository.org/).

### Study approval.

All subjects gave written informed consent and research was conducted in compliance with all relevant ethical regulations. Ethical approval was given by the East Liverpool NHS Research and Ethics Committee (REC), reference numbers 17/NW/0029 and 14/NW/1460.

## Author contributions

SPJ contributed to conceiving, designing, conducting, and analyzing experiments, design of the study, and writing of the manuscript. KR, CS, and AV contributed to designing, conducting, and analyzing experiments. SBG, LL, J. Rylance, AMC, and SCL contributed to conceiving and designing the study. EM, EN, BFC, AS, SP, ELG, J. Reine, CMW, and PFD contributed to conducting and analyzing experiments. HH, RR, ADHW, SCL, and MDW contributed to sample collection. RSH, HHS, BCU, and MY contributed to designing and analyzing experiments. DMF contributed to conceiving, designing, and analyzing experiments, design of the study, and writing of the manuscript. All authors have read and approved the manuscript. The authorship order between shared fist and shared senior authors was decided alphabetically.

## Supplementary Material

Supplemental data

Supplemental Video 1

## Figures and Tables

**Table 2:**
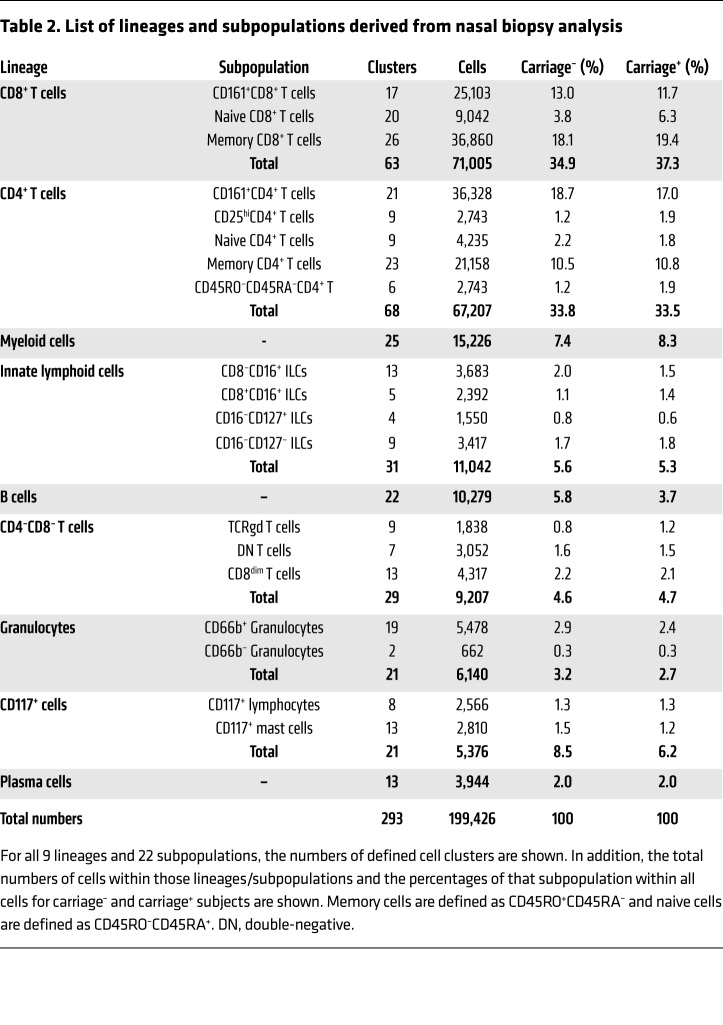
List of lineages and subpopulations derived from nasal biopsy analysis

**Table 1:**
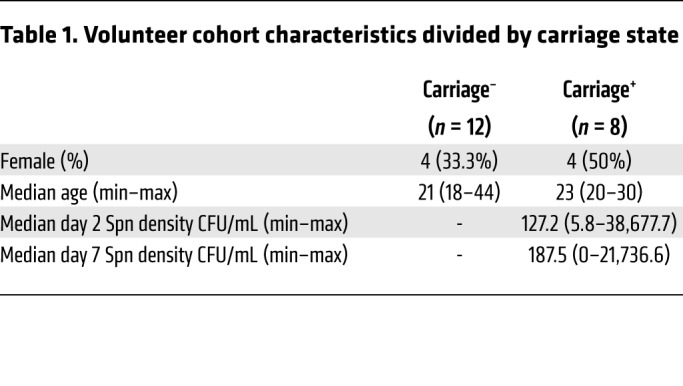
Volunteer cohort characteristics divided by carriage state

## Version 1 07/30/2019

In-Press Preview

## Version 2 09/16/2019

Electronic publication

## Version 3 10/01/2019

Print issue publication
