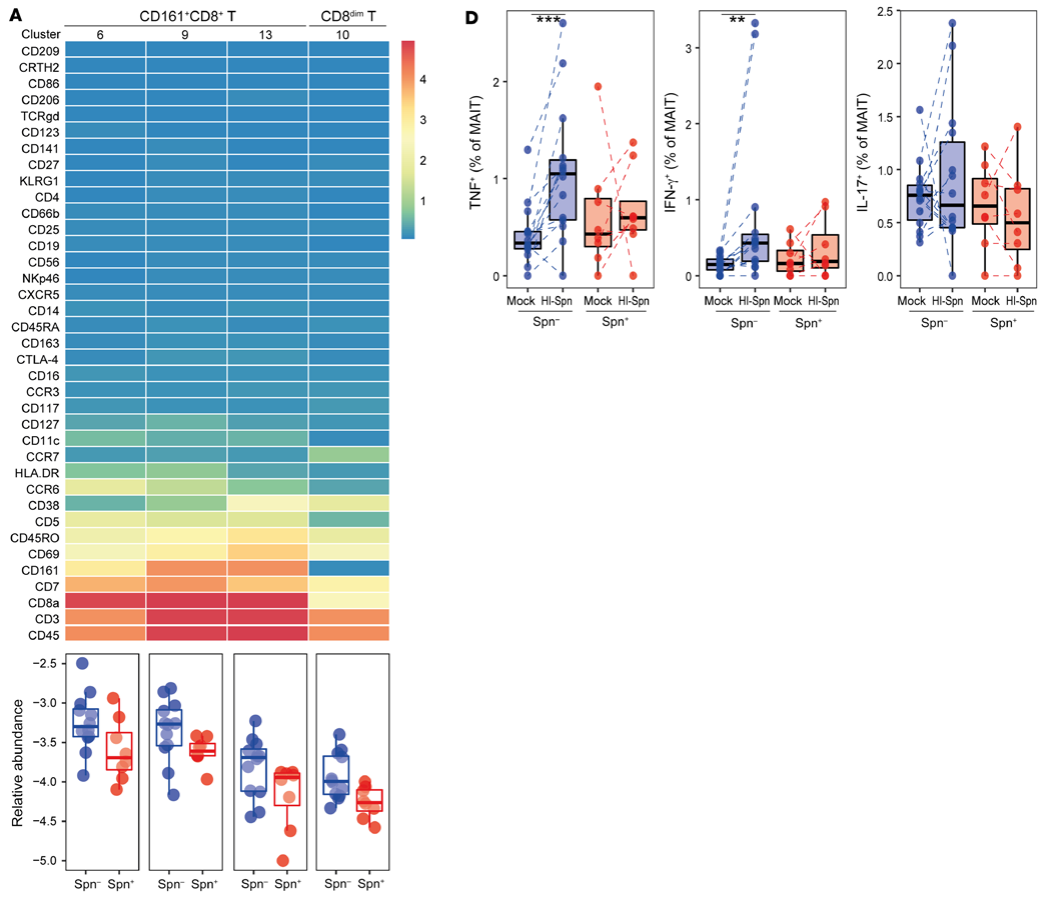# Innate and adaptive nasal mucosal immune responses following experimental human pneumococcal colonization

**DOI:** 10.1172/JCI161565

**Published:** 2022-06-01

**Authors:** Simon P. Jochems, Karin de Ruiter, Carla Solórzano, Astrid Voskamp, Elena Mitsi, Elissavet Nikolaou, Beatriz F. Carniel, Sherin Pojar, Esther L. German, Jesús Reiné, Alessandra Soares-Schanoski, Helen Hill, Rachel Robinson, Angela D. Hyder-Wright, Caroline M. Weight, Pascal F. Durrenberger, Robert S. Heyderman, Stephen B. Gordon, Hermelijn H. Smits, Britta C. Urban, Jamie Rylance, Andrea M. Collins, Mark D. Wilkie, Lepa Lazarova, Samuel C. Leong, Maria Yazdanbakhsh, Daniela M. Ferreira

Original citation: *J Clin Invest*. 2019;129(10):4523–4538. https://doi.org/10.1172/JCI128865

Citation for this erratum: *J Clin Invest*. 2022;132(11):e161565. https://doi.org/10.1172/JCI161565

During the preparation of this manuscript, the labels for Figure 5, A and D, for *Streptococcus pneumoniae* (Spn) carriage^–^ (Spn^–^) and carriage^+^ (Spn^+^) were inverted. The correct figure panels are below. The HTML and PDF versions of the figure have also been updated.

The *JCI* regrets the errors.

## Figures and Tables

**Figure:**