# Supplementary material for: Innate and adaptive nasal mucosal immune responses following experimental human pneumococcal colonization
Source: J Clin Invest. 2019 Sep 16;129(10):4523–38. doi: 10.1172/JCI128865 (PMC6763269; doi:10.1172/JCI128865)
Supplement: Supplemental data [file jci-129-128865-s273.pdf]

Supplementary Table 1. List of antibodies used for CyTOF.

#	Label	Specificity	Clone	Provider	Catalogue
**1**	^{89}Y	CD45	HI30	Fluidigm	3089003B
**2**	^{115}La	CD5	UCHT2	BioLegend	300627
**3**	^{141}Pr	CD196 (CCR6)	G034E3	Fluidigm	3141003A
**4**	^{142}Nd	CD19	HIB19	Fluidigm	3142001B
**5**	^{143}Nd	CD117 (c-Kit)	104D2	Fluidigm	3143001B
**6**	^{144}Nd	CD66b	REA306	Miltenyi	130-108-019
**7**	^{145}Nd	CD4	RPA-T4	Fluidigm	3145001B
**8**	^{146}Nd	CD8a	RPA-T8	Fluidigm	3146001B
**9**	^{148}Nd	CD14	M5E2	BioLegend	301843
**10**	^{149}Sm	CD25 (IL-2Ra)	2A3	BioLegend	301843
**11**	^{150}Nd	CD185 (CXCR5)	J252D4	BioLegend	356902
**12**	^{151}Eu	CD123	6H6	Fluidigm	3151001B
**13**	^{152}Sm	TCRγδ	11F2	Fluidigm	3152008B
**14**	^{153}Eu	CD7	CD7-6B7	Fluidigm	3153014B
**15**	^{154}Sm	CD163	GHI/61	Fluidigm	3154007B
**16**	^{155}Gd	CD69	FN50	BioLegend	310939
**17**	^{156}Gd	CD294 (CRTH2)	BM16	BioLegend	350102
**18**	^{158}Gd	CD209	9E9A8	BioLegend	330102
**19**	^{159}Tb	CD197 (CCR7)	G043H7	Fluidigm	3159003A
**20**	^{161}Dy	KLRG1 (MAFA)	REA261	Miltenyi	Special order
**21**	^{162}Dy	CD11c	Bu15	Fluidigm	3162005B
**22**	^{163}Dy	CD152 (CTLA-4)	BNI3	BioLegend	369602
**23**	^{164}Dy	CD161	HP-3G10	Fluidigm	3164009B
**24**	^{165}Ho	CD127 (IL-7Ra)	AO19D5	Fluidigm	3165008B
**25**	^{166}Er	CD141	AD5-14H12	Miltenyi	130-108-033
**26**	^{167}Er	CD27	O323	Fluidigm	3167002B
**27**	^{168}Er	HLA-DR	L243	BioLegend	307651
**28**	^{169}Tm	CD45RA	HI100	Fluidigm	3169008B
**29**	^{170}Er	CD3	UCHT1	Fluidigm	3170001B
**30**	^{171}Yb	CD206	15-2	BioLegend	321127
**31**	^{172}Yb	CD38	HIT2	Fluidigm	3172007B
**32**	^{173}Yb	CD45RO	UCHL1	BioLegend	304239
**33**	^{174}Yb	CD335 (NKp46)	92E	BioLegend	331902
**34**	^{175}Lu	CD193 (CCR3)	5E8	Fluidigm	3175025B
**35**	^{176}Yb	CD56	NCAM16.2	Fluidigm	3176008B
**36**	^{194}Pt	Epcam	9C4	BioLegend	324229
**37**	^{198}Pt	CD86	IT2.2	BioLegend	305435
**38**	^{209}BI	CD16	3G8	Fluidigm	3209002B

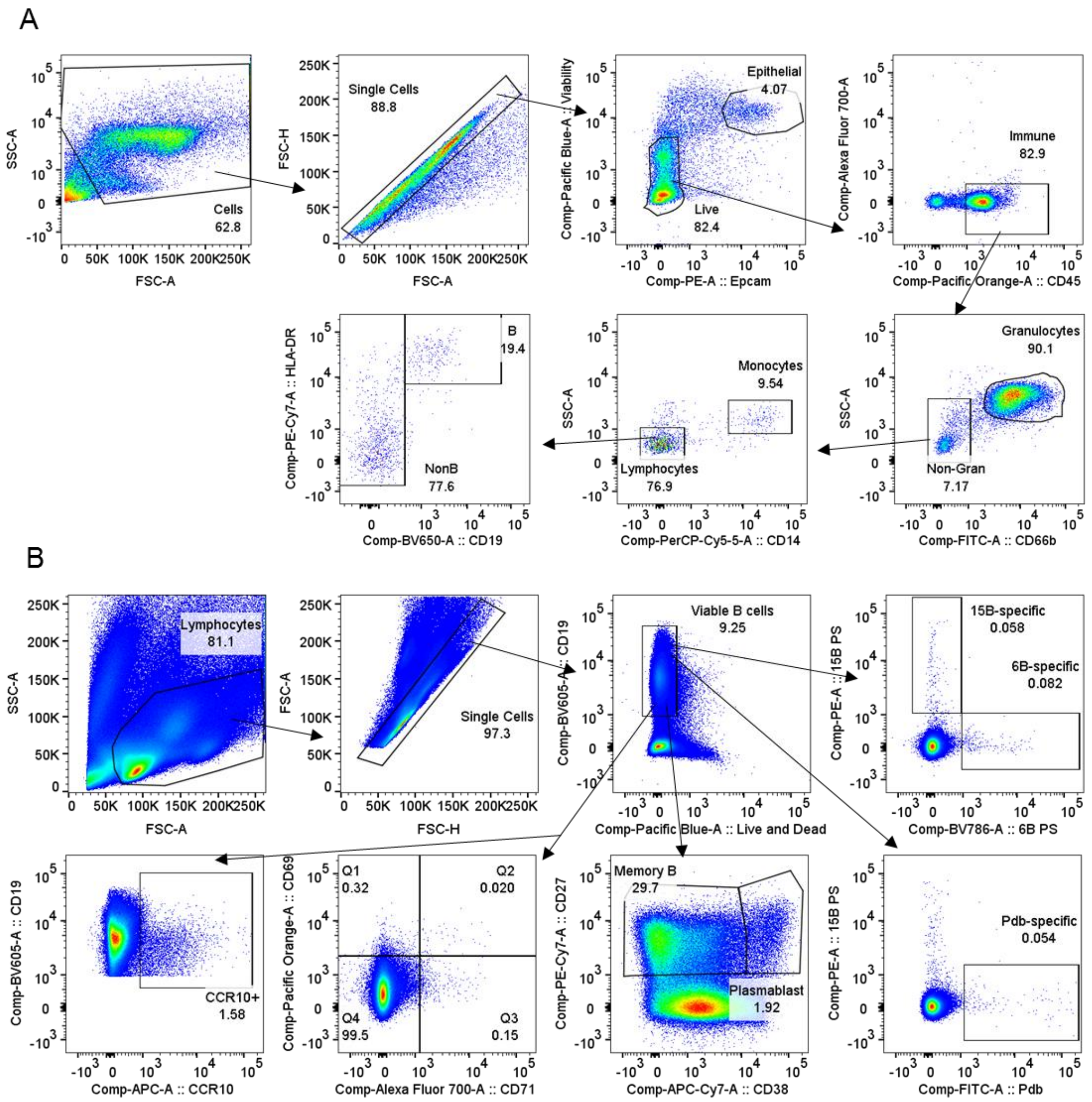

Supplementary Figure 1. B cell validation flow cytometry gating strategies. A) Gating strategy for nasal CD19$^+$ B cells for a representative nasal curette sample. Each plot shows the cells contained in the precedent gate. Population names and frequencies are shown. B) Gating strategy for the detection of pneumococcus-specific B cells. Each plot shows the cells contained in the precedent gate. Population and frequency are shown. Gates were set on all B cells and copied into Spn-specific populations.

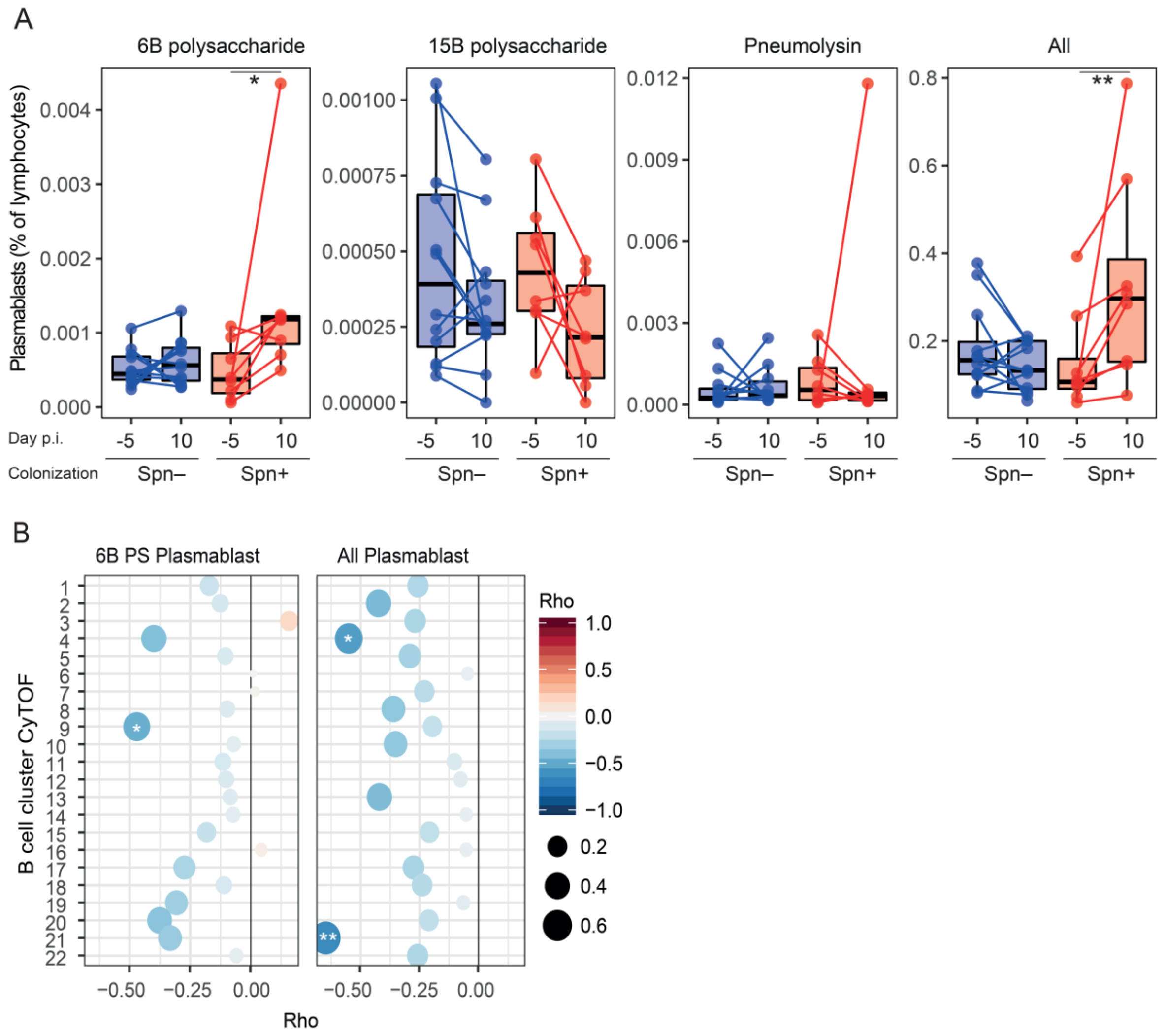

Supplementary Figure 2. Normalization of circulating plasmablasts to total lymphocyte numbers. A) Levels of 6B polysaccharide-specific, 15B polysaccharide-specific, Pneumolysin derivative b (Pneumolysin)-specific or all plasmablast amongst total lymphocytes were measured from PBMC collected at baseline (Day -5) and at the time of biopsy (Day 10 post inoculation). Boxplots and individual subjects are depicted with carriage– in blue (n=12) and carriage+ in red (n=8), with paired samples connected by dashed lines. * $p < 0.05$, ** $p < 0.01$ by Wilcoxon test comparing a group to its baseline. B) Correlations between fold-change in levels of 6B PS-specific and total plasmablasts between baseline and day 10 normalized against total number of lymphocytes against levels of B cell clusters measured by CyTOF. Color and size of symbols reflect the Spearman rho value. * $p < 0.05$ and ** $p < 0.01$ by Spearman test.

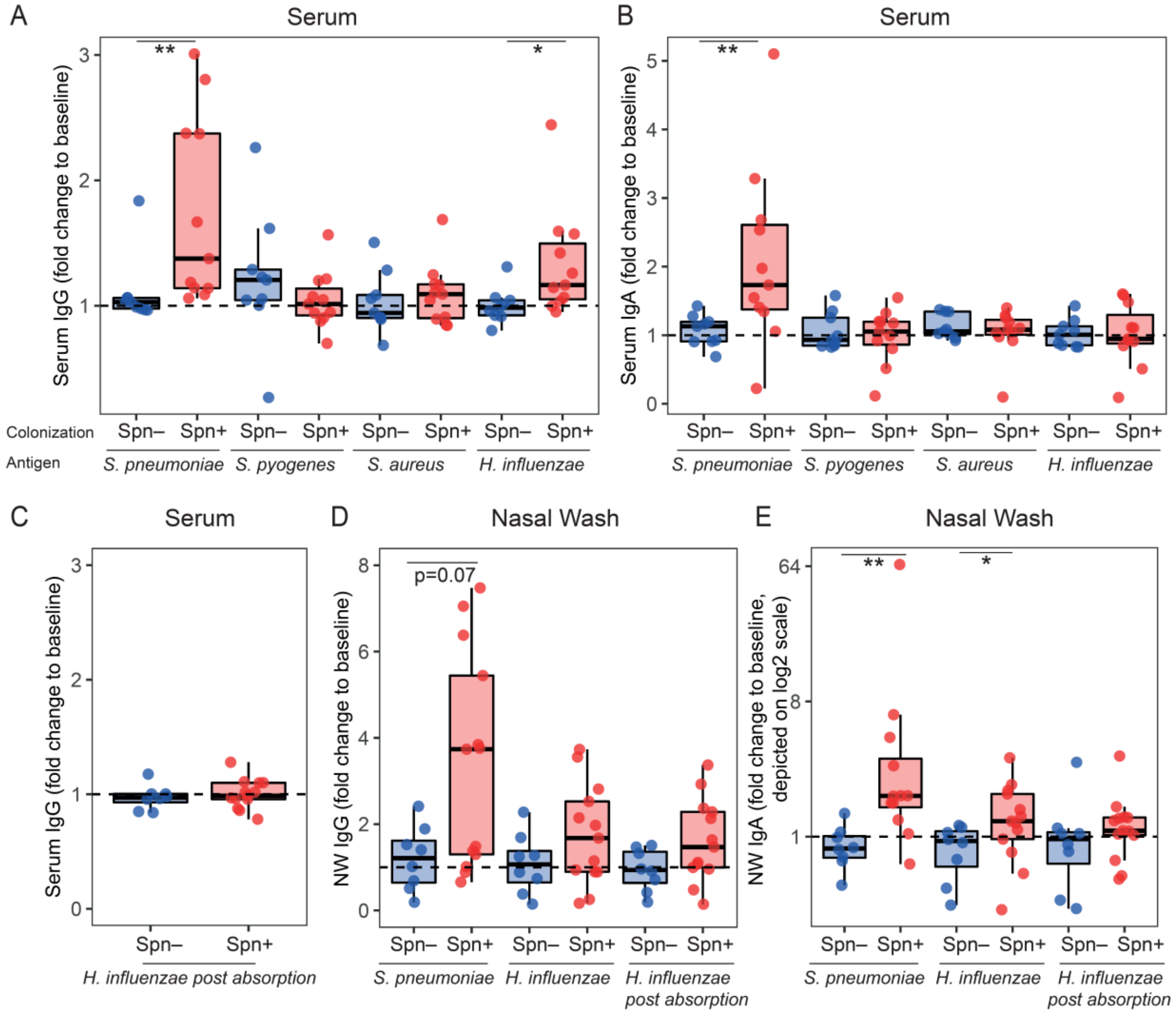

Supplementary Figure 3. Antibody levels against respiratory pathogens. Fold change (day 23 post inoculation versus baseline) in serum levels of A) IgG and B) IgA against *Streptococcus pneumoniae*, *Streptococcus pyogenes*, *Staphylococcus aureus* and *Haemophilus influenzae*. C) Fold change of serum levels of *Haemophilus influenzae*-specific IgG post absorption with Spn. Nasal wash (NW) levels of D) IgG and E) IgA against *Streptococcus pneumoniae* and *Haemophilus influenzae* (with or without pre-absorption with Spn). Boxplots and individual subjects are depicted with carriage^{-} in blue (Spn–, n=8-9) and carriage+ in red (Spn+, n=11-13). * $p < 0.05$, ** $p < 0.01$ by Mann-Whitney test comparing fold-change levels between carriage^{-} and carriage^{+} subjects.

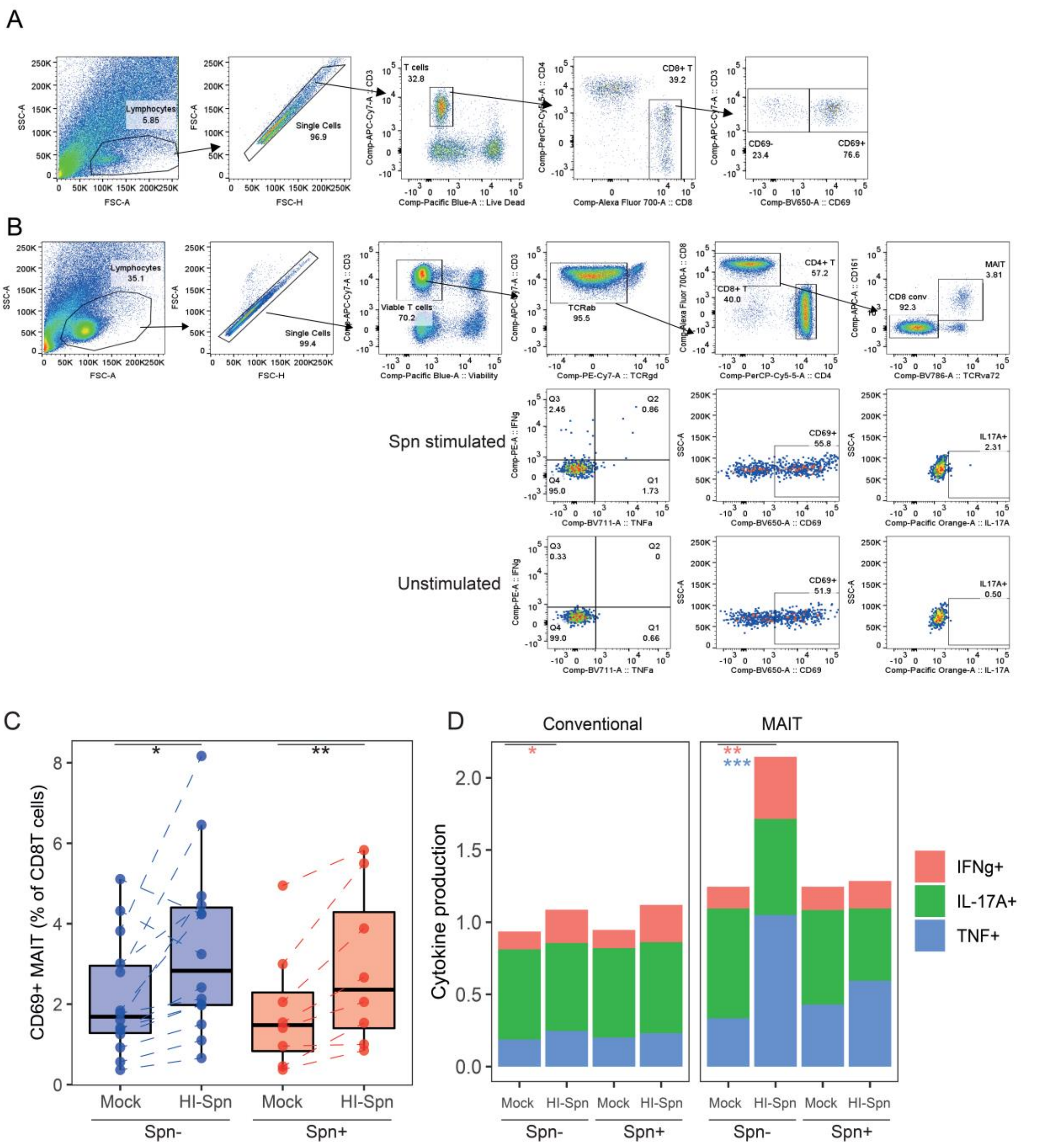

Supplementary Figure 4. CD8^{+} T cell flow cytometry gating strategies. A) Gating strategy for CD8^{+} T tissue-resident memory cells by flow cytometry for a representative nasal biopsy. Each plot shows the cells contained in the precedent gate. Population and frequency are shown. B) Gating strategy to detect CD8^{+} mucosal associated invariant T cells in PBMC. One representative heat-inactivated pneumococcus (HI-Spn)-stimulated sample is depicted. For cytokine production and CD69 activation large dots are used to better show rare events. Each plot shows the cells contained in the precedent gate. Population and frequency are shown. C) Levels of CD69 on MAIT cells after stimulation or not. Individuals are shown and connected by lines and boxplots are overlaid. D) Stacked bar charts showing the median level of cytokine

production (IFN-ɣ in red, IL-17A in green and TNF in blue) for conventional and MAIT CD8+ T cells. *p<0.05, **p<0.01, ***p<0.001 by Wilcoxon test.

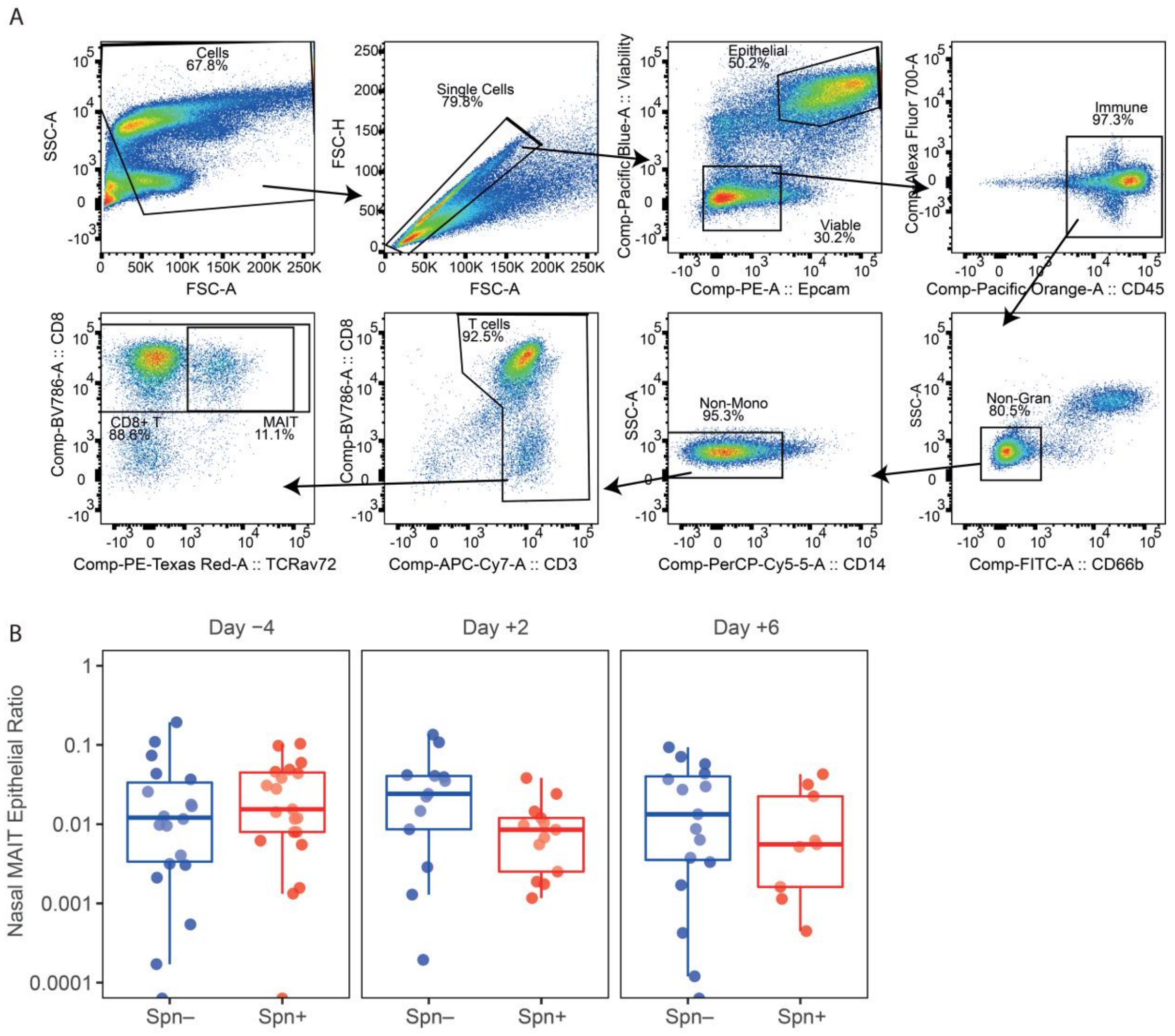

Supplementary Figure 5. Nasal CD8⁺ mucosal associated invariant T cell measured longitudinally following pneumococcal challenge. A) Gating strategy for a representative nasal curette sample is shown. Each plot shows the cells contained in the precedent gate. Population and frequency are shown. B) Boxplots and individual subjects are depicted prior to Spn challenge and at days 2 and 6 post challenge. Spn– subjects are depicted in blue (n=19) and Spn+ subjects in red (n=20).

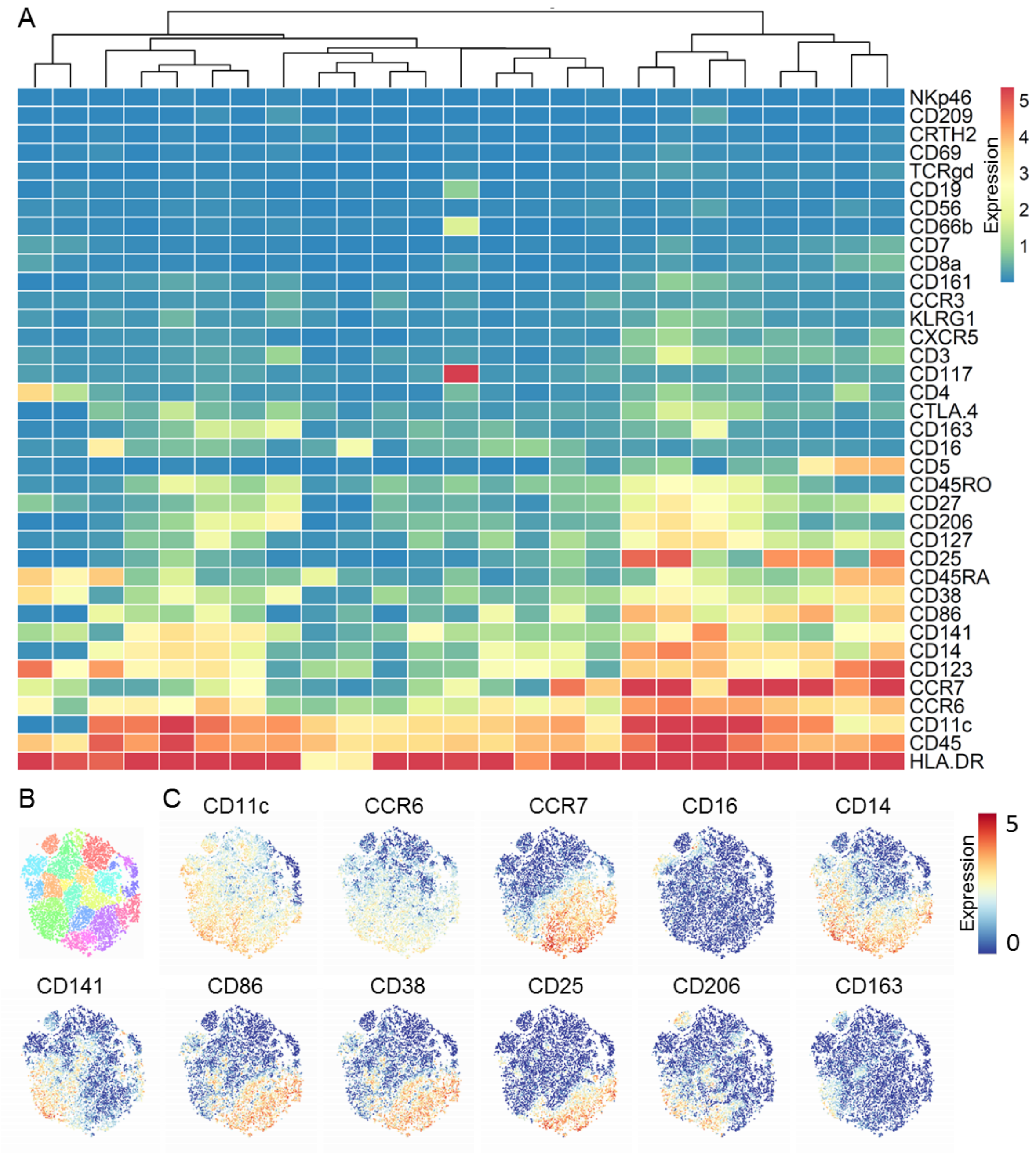

Supplementary Figure 6. Nasal monocytes/macrophages are predominantly CD14$^+$ CD16$^–$ classical monocytes. A) Heatmap showing for each of the monocyte clusters (columns) the expression for thirty-seven markers (rows). Markers are ordered by increasing median expression. Columns are re-ordered and a cluster dendrogram is shown. B) Cluster definition within the myeloid cell lineage. C) Fingerprint graphs showing expression for selected markers on a single-cell level.

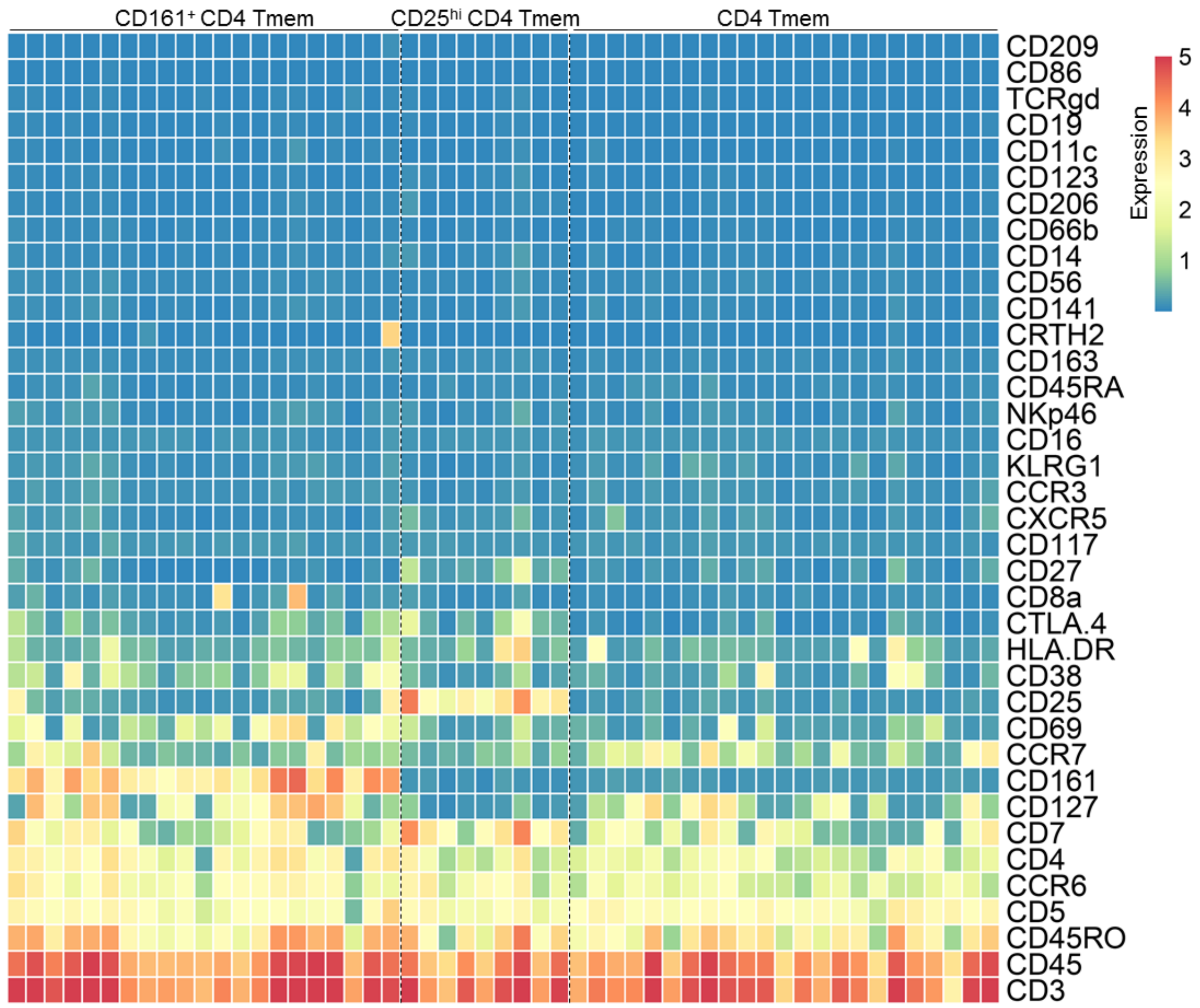

Supplementary Figure 7. Nasal memory CD4 T cell phenotype. Heatmap including each of the three memory (CD45RO+) CD4+ T cell subpopulations within the CD4+ T cell lineage. All identified clusters (columns) with expression for thirty-seven markers (rows) are shown. Clusters belonging to the CD161^{+}, CD25^{hi} and CD4 T memory (Tmem) subpopulations are separated by dashed lines. Markers are ordered by increasing median expression.

Supplementary Table 2. List of antibodies used for flow cytometry

#	Fluorochrome	Specificity	Clone	Provider	Catalogue
**1**	PE/TxsRed	TCRVa7.2	3C10	Biolegend	351730
**2**	PerCP/Cy5.5	CD14	MφP9	BD Biosciences	562692
**3**	BV650	CD19	HIB19	Biolegend	302238
**4**	APC/Cy7	CD3	SK7	Biolegend	344818
**5**	BV510	CD45	HI30	Biolegend	304036
**6**	PacificOrange	CD45	HI30	Thermofisher	MHCD-4530
**7**	PECy7	HLADR	L243	Biolegend	307616
**8**	PE	EpCAM	9C4	Biolegend	324206
**9**	BV785	CD8	SK1	Biolegend	344740
**10**	FITC	CD66b	G10F5	Biolegend	305104
**11**	BV711	TCRVa7.2	3C10	Biolegend	351732
**12**	APC	CD161	HP-3G10	Biolegend	339912
**13**	BV650	CD69	FN50	Biolegend	310934
**14**	PE/Dazzle594	CD25	M-A251	Biolegend	356126
**15**	BV605	CD103	Ber-ACT8	Biolegend	350218
**16**	PerCP/Cy5.5	CD45	HI30	Biolegend	304028
**17**	AF700	CD8	SK1	Biolegend	344724
**18**	BV785	TCRVa7.2	3C10	Biolegend	351722
**19**	APC/H7	CD3	SK7	BD Biosciences	560176
**20**	PE/Cy7	TCRgd	11F2	BD Biosciences	655410
**21**	AF488	FOXP3	259D	Biolegend	320212
**22**	PE	IFNg	4S.B3	Biolegend	502509
**23**	BV711	TNFa	MAb11	Biolegend	502940
**24**	BV510	IL17a	N49-653	BD Biosciences	563295
**25**	AF700	CD71	M-A712	BD Biosciences	563769
**26**	BV605	CD19	SJ25C1	BD Biosciences	562653
**27**	PE/Cy7	CD27	M-T271	BD Biosciences	560609
**28**	APC/Cy7	CD38	HIT2	Biolegend	303534
**29**	BV510	CD69	FN50	Biolegend	310936
**30**	APC	CCR10	1B5	BD Biosciences	564771
**31**	PE/TxsRed	TCRVa7.2	3C10	Biolegend	351730

Supplementary Table 3. List of antibodies used for immunohistochemistry.

#	Antibody	Specificity	Clone	Provider	Catalogue
**1**	Primary	CD3	F7.2.38	Dako	M7254
**2**	Primary	CD4	EPR6855	Abcam	ab133616
**3**	Primary	CD20	EP459Y	Abcam	ab78237
**4**	Primary	CD66b	polyclonal	Abcam	ab214175
**5**	Primary	CD68	EPR20545	Abcam	ab213363
**6**	Primary	CD11b	EP1345Y	Abcam	ab52478
**7**	Primary	CD8	EP1150Y	Epitomics	1872-1
**8**	Primary	CD161	polyclonal	Atlas antibodies	HPA039113
**9**	Peroxidase	a-rabbit		Vector labs	PI-1000
**10**	Peroxidase	a-mouse		Vector labs	MP-7452
